# Advances in breeding for high grain Zinc in Rice

**DOI:** 10.1186/s12284-016-0122-5

**Published:** 2016-09-26

**Authors:** B. P. Mallikarjuna Swamy, Mohammad Akhlasur Rahman, Mary Ann Inabangan-Asilo, Amery Amparado, Christine Manito, Prabhjit Chadha-Mohanty, Russell Reinke, Inez H. Slamet-Loedin

**Affiliations:** 1 Plant Breeding, Genetics, and Biotechnology Division, International Rice Research Institute (IRRI), DAPO Box 7777, Metro Manila, Philippines; 2 Plant Breeding Division, Bangladesh Rice Research Institute (BRRI), Gazipur, Bangladesh

**Keywords:** Biofortification, Zinc, Breeding, Marker, QTL, Gene

## Abstract

Zinc (Zn) is one of the most essential micronutrients required for the growth and development of human beings. More than one billion people, particularly children and pregnant women suffer from Zn deficiency related health problems in Asia. Rice is the major staple food for Asians, but the presently grown popular high yielding rice varieties are poor supplier of Zn in their polished form. Breeding rice varieties with high grain Zn has been suggested to be a sustainable, targeted, food-based and cost effective approach in alleviating Zn deficiency. The physiological, genetic and molecular mechanisms of Zn homeostasis have been well studied, but these mechanisms need to be characterized from a biofortification perspective and should be well integrated with the breeding processes. There is a significant variation for grain Zn in rice germplasm and efforts are being directed at exploiting this variation through breeding to develop high Zn rice varieties. Several QTLs and gene specific markers have been identified for grain Zn and there is a great potential to use them in Marker-Assisted Breeding. A thorough characterization of genotype and environmental interactions is essential to identify key environmental factors influencing grain Zn. Agronomic biofortification has shown inconsistent results, but a combination of genetic and agronomic biofortification strategies may be more effective. Significant progress has been made in developing high Zn rice lines for release in target countries. A holistic breeding approach involving high Zn trait development, high Zn product development, product testing and release, including bioefficacy and bioavailability studies is essential for successful Zn biofortification.

**Electronic supplementary material:**

The online version of this article (doi:10.1186/s12284-016-0122-5) contains supplementary material, which is available to authorized users.

## Introduction

Micronutrient deficiencies or hidden hunger has become a major nutritional problem affecting more than two billion people in the developing countries of Asia, Africa, and Latin America. Also, the micronutrient malnutrition associated health risks have become a major hindrance in achieving the Millennium Development Goals (MDG) such as reducing poverty and hunger, improved maternal health status, and less child mortality (Cakmak 2008; White and Broadley 2011; Wessells and Brown 2012) and these are also important sustainable development goals (SDGs) to be achieved by 2035 (https://sustainabledevelopment.un.org).

Zinc (Zn) is one of the essential micronutrients, which serves as a co-factor for more than 300 enzymes involved in the metabolism of carbohydrates, lipids, proteins, and nucleic acids, hence its importance in normal growth and development of plants and animals (Keith et al. 2006; Roohani et al. 2013; Sadeghzadeh 2013). One-third of the human population, particularly children and women suffer from Zn deficiency related health problems such as growth retardation, loss of appetite, impaired immune function, hair loss, diarrhea, eye and skin lesions, weight loss, delayed healing of wounds, and mental lethargy (Hotz and Brown 2004; Institute of Medicine Food and Nutrition Board IMFNB 2001; Prasad 2004; Wang and Busbey 2005). Some of these problems are more acute and clearly evident in developing countries where people depend on cereal-based foods for their daily diet and they cannot afford to diversify their meal by adding mineral-rich fruits, vegetables, and meat (Maret and Sandstead 2006; Shahzad et al. 2014).

An adequate supply of Zn is essential for maintaining a healthy and productive life, and the average daily requirement for Zn is 7–13 mg per day for adults (Department of Health (UK) 1991; Institute of Medicine Food and Nutrition Board IMFNB 2001). A change of diet to Zn-rich food, preventive supplementation of Zn, and Zn fortification of processed foods are all being used to reduce human Zn-deficiency related problems, but these approaches have limited impact because of the recurring costs and also the ineffective delivery systems (Berti et al. 2014). Biofortification of staple food crops with Zn has been suggested to be an alternative, complementary, and sustainable approach to overcome Zn malnutrition, as staple foods are eaten in large quantities on a daily basis by malnourished poor (Thorne-Lyman et al. 2010; Bouis and Welch 2010).

Rice is the major staple food and source of energy for more than half of the world’s population, but the presently grown popular high yielding rice varieties are a poor source of essential micronutrients such as Zn in their polished (white) form (Kennedy et al. 2002; Sharma et al. 2013). The biofortification of rice with enhanced levels of Zn in its polished form may be a cost-effective and sustainable solution to assist in combating Zn malnutrition.

Over the last decade, several efforts have been made to biofortify food crops with micronutrients, which led to a significant understanding of the physiological, genetic, and molecular basis of high Zn accumulation in grains, and also the influence of agronomic management and environmental factors on Zn uptake, translocation and loading into grains (Impa and Johnson-Beebout 2012). Several genetic studies have also been carried out to identify Quantitative Trait Loci (QTLs) for high Zn in grains, and there is a great potential to use them in marker assisted breeding. Candidate genes involved in Fe and Zn uptake and accumulation have also been identified in rice and successfully used in developing high Zn transgenic lines. Breeding efforts could increase the Zn level by 6–8 mg kg ^−1^ (HarvestPlus 2014); while transgenic rice lines developed show an improvement of 15–30 mg kg ^−1^ in Zn levels (Johnson et al. 2011; Masuda et al. 2012; Slamet-Loedin et al. 2015). However, deregulation of Genetically Modified (GM) products for cultivation is still a major challenge. In our review, we discuss the recent advances in the physiological, genetic, and molecular basis of high grain Zn, approaches for biofortification of Zn, advances in breeding for high Zn rice, status of high Zn rice product development and delivery in the target countries.

## Review

### Physiological basis of grain Zn

In order to accumulate Zn in grains, rice plants have to uptake, mobilize, and transport Zn from soil to grain, which involves many complex physiological processes at different levels within the rice plant. Provided there is an adequate supply of Zn in the soil, biofortified rice genotypes to be developed should have the genetic potential and physiological efficiency to utilize the available Zn from the soil. A better understanding of the physiological basis of Zn uptake, its translocation, the maintenance of Zn homeostasis, Zn partitioning within and between different plant parts and within rice grain, internal allocation, re-allocation, re-mobilization, and efficient loading into grain is essential for genetic biofortification of rice, but a complete knowledge of these processes in rice is still lacking (Stomph et al. 2009; Olsen and Palmgren 2014).

In general there are three major rate limiting steps or barriers for efficient Zn accumulation in rice grain: 1) soil-to-root barriers; 2) root-to-shoot barriers; and 3) barriers in loading Zn into grains.

Root uptake is the first step towards the accumulation of Zn in rice grains. Plant factors that affect root Zn uptake include root architecture, root hairs, crown root development, root surface area, root anatomical structures and modification of rhizosphere chemistry through exudation of protons, which can change soil pH, thereby improve the solubility of Zn in the soil and facilitate its diffusion to the root surface (Rose et al. 2013). Soil factors that affect the plant-availability of Zn for all crops include soil pH, texture, organic matter content, mineralogy, and microbial populations (Hacisalihoglu and Kochian 2003; White and Broadley 2011). The availability of soil Zn for rice from flooded (anaerobic) soil is affected by an additional set of factors including soil redox potential, total sulfur content, and soluble bicarbonate (Impa and Johnson-Beebout 2012). Thus, a combination of agronomic management practices and genetic approaches are essential to improve the soil health conditions to enhance the root uptake of Zn.

In rice, direct root uptake, remobilizations from vegetative tissues or combination of both of these two approaches are the main source of Zn in grains (Impa et al. 2013a). There is a continuous xylem flow from root to grain mediated by transpiration system, which can directly transport Zn to grains (Krishnan and Dayanandan 2003); however Zn movement is restricted by the presence of barriers for root-to-shoot transfer and for internal allocation and re allocation of Zn within and between vegetative and reproductive tissues, which leads to reduced accumulation of Zn in grains (Jiang et al. 2008). Suberin in the cell wall, casparian strips, Zn sequestration in cytoplasm and vacuoles, and anatomical variations in root-shoot junction are some of the root-to-shoot barriers for Zn transport (Yamaguchi et al. 2012; Yamaji et al. 2013). The Zn taken up by roots is translocated to different plant parts by xylem and phloem, and there is a huge variation in Zn allocation and reallocation between different organs, tissues and cells of root and shoot (Jiang et al. 2008). However, genotypic differences exist in loading of Zn in xylem and phloem and its unloading into different tissues (Jiang et al. 2008), which clearly indicates that by breeding, there is a possibility to improve the efficiency of root uptake, root-to-shoot translocation and internal allocation of Zn.

Efficient loading of Zn into grains, especially to the endosperm is most important for Zn biofortification of rice (Waters and Sankaran 2011). Eventhough there are huge amounts of Zn in vegetative tissues of rice plants, remobilization of Zn from vegetative tissues to reproductive tissues and finally to grains is limited due to selective phloem transport of Zn from old tissues to new tissues and to the grains (Wu et al. 2010; Impa et al. 2013a). Flag leaf, which plays an important role in photosynthesis and grain yield, was found to have a little contribution to grain Zn (Sperotto et al. 2013), while Wu et al. (2010) reported significant translocation of Zn from flag leaf to the grain. A continuous supply of Zn to different tissues throughout the life cycle by translocation and phloem remobilization to grains is an important feature of Zn efficient rice genotypes (Yin et al. 2016). Through transgenic approaches and over expression of Zn homeostasis genes such as *OsZIP1, OsZIP4*, *OsZIP8*, *OsZIP8a*, *OsYSL8, OsYSL9 OsFRO2A, OsNAS1*, *OsNAS2*, *OsNAS3, OsArd2*, *OsIRT1*, *OsNRAMP1* and *OsHMA2* several studies have highlighted the importance of efficient Zn uptake and unhindered transportation of Zn among different plant tissues especially during grain filling stages (Ishimaru et al. 2005, 2007, 2011; Chandel et al. 2010; Yamaji et al. 2013; Sasaki et al. 2014). It is also interesting to note that at lower tissue Zn concentrations, most of the Zn was found in leaf and reproductive tissues, while at higher Zn levels, stem and roots showed increased Zn. Also, the increased root uptake of Zn and root to shoot transfer could not proportionately increase the grain Zn concentrations indicating that internal translocation/retranslocation of Zn from vegetative tissues to grains is the major bottleneck for improving grain Zn concentrations (Stomph et al. 2014; Yin et al. 2016).

Though, a number of physiological studies have been published about Zn-efficient rice, little is known on how Zn is redistributed and remobilized from vegetative tissues to the grains (Ren et al. 2006). A better understanding of the mechanisms involved in loading of Zn into the endosperm of rice and identification of rice genotypes with better Zn remobilization capacity without having any adverse effect on yield will be highly useful for Zn biofortification of rice (Jiang et al. 2007; Wu et al. 2010). Rice has also been found to show different levels and patterns of Zn accumulation under high or low Zn conditions and in different rice ecosystems (Wissuwa et al. 2006; Mabesa et al. 2013; Impa et al. 2013b).

### Genetic basis of grain Zn

Increasing the bioavailable Zn in the rice endosperm is the major goal of rice biofortification. There is a variation in the pattern of Zn distribution within rice grain with the aleurone layer having 25–30 % of the total Zn, and this is lost during processing, while the endosperm has 60–75 % of Zn, which is retained even after polishing (Hansen et al. 2009). The genetic basis of high grain Zn in brown/polished rice is very complex and a better understanding of the genetic basis of high grain Zn in rice is essential for the systematic utilization of rice germplasm in Zn biofortification programs. Grain Zn has a moderate to high broad-sense heritability and can be improved by breeding (Norton et al. 2010; Zhang et al. 2014), while reports of narrow sense heritability clearly indicated significant additive and dominant genetic effects. Also, grain Zn has been found to be significantly influenced by the environmental factors (Gregorio 2002; Chandel et al. 2010; Anuradha et al. 2012a). Genetic characterization of grain Zn in several Recombinant Inbred Lines (RILs) and also in rice germplasm collections has shown significant Phenotypic Co-efficient of Variation (PCV), Genotypic Co-efficient of Variation (GCV), broad-sense Heritability and Genetic Advance (GA) (Table 1). In 12 out of the 14 studies, biparental mapping populations were used and in two studies germplasm collections were used for genetic characterization of Zn concentrations. One population was derived from wild progenitor species *O. rufipogon*. Among the different studies PCV and GCV for grain Zn concentration varied from 9.3 % to 40 % and from 9.2 % to 36 % respectively, while heritability varied from 41 % to 99.4 % and GA varied from 18.6 % to 66.6 %. Highest PCV and GCV values were reported in Azucena × Moromutant population, while lowest in TRY (R) 2 × Mapillaisamba population. Heritability and genetic advance were highest in BPT5204 × HPR14 and Azucena x Moromutant populations respectively. All these results show that there is a sufficient variation for grain Zn concentration with moderate to high heritability and genetic advance. Thus it is possible to improve the Zn concentration of popular rice varieties by exploiting high Zn gemplasm in the breeding programs.

**Table 1:** Genetic parameters for grain Zinc concentration in rice

S. No	Population	PCV(%)	GCV(%)	Heritability (%)	Genetic advance (% mean)	Reference
1	ADT 37 × IR68144-3B-2-2-3	19.2	18.6	94.2	37.2	Sala et al. 2013
2	ADT 43 × IR68144-3B-2-2-3	15.6	15.2	94..1	30.4	Sala et al. 2013
3	TRY (R) 2 × Mapillaisamba	9.3	9.2	96.8	18.6	Sala et al. 2013
4	TRY (R) 2 × IC 255787	17.2	17.0	98.0	34.8	Sala et al. 2013
5	Rice land races	21.9	18.4	70.6	31.9	Thongbam et al. 2012
6	Rice hybrids	11.7	10.8	85.8	20.7	Babu et al. 2012
7	BPT5204 × HPR14	26.1	26.0	99.4	53.6	Samak et al. 2011
8	Rice genotypes	25.5	21.1	94.0	30.1	Bekele et al. 2013
9	IRRI38 × Jeerigesanna	18.4	17.0	85.6	32.5	Gande et al. 2013
10	F_2_ population	-	-	96.9	-	Zhang et al. 2004
11	BIL mapping population	10.8	-	76.4	-	Susanto 2008
12	Azucena × Moromutant	40.1	36	80.6	66.6	Bekele et al. 2013
13	Bala × Azucena	-	-	>60	-	Norton et al. 2010
14	Teqing × *O rufipogon*	-	-	41	-	Garcia-Oliveira et al. 2009

The combining ability analysis by diallel crosses involving seven specific rice varieties with different levels of Zn showed that additive genetic effects were more important for Zn, while the co-efficient of variation (CV) for Zn varied significantly among the entries over the years and locations, indicating significant genotype and environment interactions (G x E) (Zhang et al. 1996; Sharifi 2013). In another study involving black pericarp *indica* rice, genetic and cytoplasmic effects influenced the final grain Zn content, but the genetic effect was stronger and it constituted the major portion of the seed genetic effects. The heritability of the seed genetic effect was highly significant and narrow-sense heritability was very high, suggesting single plant selection as an effective approach for improving Zn content. There is also a positive correlation between grain Zn and the grain characteristics such as grain weight, grain length and width, so during the selection process, some consideration should be given to grain traits (Zhang et al. 2004). However, in a RIL population platykurtic and skewed distributions were observed for grain Zn, indicating involvement of several minor genes with duplicate gene interactions indicating little improvement by direct selection (Banu and Jagadeesh 2014).

Significant positive heterosis for grain Zn has also been reported. In a line × tester analysis involving six lines and eight testers and a total of 48 hybrids, it was interesting to note that 14 out of 48 hybrids showed significant positive heterosis for grain Zn over the standard micronutrient check variety Chittimutyalu. Two crosses (PR116 × Chittimutyalu, Mandya Vijay × Jalamagna) showed more than 50 % heterosis for grain Zn (Babu et al. 2012). Transgressive segregants were also observed for grain Zn (Stangoulis et al. 2007).

High grain Zn trait was found to be tightly linked with aroma, while there are no reports of pleiotropic effects of high grain Zn with other traits (Welch and Graham 2004; Gregorio 2002). There are several reports indicating epistatic interactions for grain Zn (Lu et al. 2008; Norton et al. 2010). In some cases, genetic factors increasing Zn also co-segregate with genetic factors that increase Fe and other mineral elements (Gregorio 2002; Jiang et al. 2007). Grain quality traits and grain Zn was also found to be correlated (Anandan et al. 2011; Zhang et al. 2004). All the associations of grain Zn with different mineral elements and grain quality traits must be taken into consideration while breeding for high Zn rice.

One of the most important aspects of high Zn rice development is the relationship between grain Zn concentration and grain yield. Several reports indicate a significant negative association between grain Zn concentration and yield in rice (Gao et al. 2006; Jiang et al. 2008; Norton et al. 2010; Wissuwa et al. 2007), but a positive relationship between grain yield and grain Zn concentration was observed under Zn-deficient soil (Gregorio 2002) and also in different panel of aromatic rice and land races under Zn sufficient conditions non significant correlations were observed between yield and grain Zn (Gangashetty et al. 2013; Sathisha 2013). This is also supported by the non significant correlations between yield and Zn in other cereal crops such as pearl millet (Rai et al. 2012). Thus, it can be concluded that it is possible develop high yielding varieties with high levels of Zn. Identification of high Zn donor lines with high yield, high Zn transgenic lines with high yield (Johnson et al. 2011; Trijatmiko et al. 2016), and recently released high Zn rice lines with high yield potential in Bangladesh provide positive evidence for the possibility of combining high Zn and high yield potential in rice (HarvestPlus 2014).

### Molecular basis of grain Zn

Identification of genes/major effect QTLs and understanding the molecular basis of grain Zn in rice will facilitate breeding for high Zn rice through Marker-Assisted Selection (MAS). Several genes/gene families involved in Zn homeostasis have been well characterized in rice (Additional file 1: Table S1). Root exudates or phytosiderophores helps in efficient release and uptake of metals from the soil (Bashir et al. 2006; Widodo et al. 2010; Nozoye et al. 2011). Some of the gene families such as *OsNAS*, *OsTOM1*, *OsDMAS*, *OsSAMS* and *OsNAAT* are involved in biosynthesis, transport and secretion of phytosiderophores in the root zone and thereby increases the metal uptake by roots (Inoue et al. 2003, 2008; Bashir et al. 2006; Widodo et al. 2010; Nozoye et al. 2011; Johnson et al. 2011). The ZIP family genes are important metal transporters found to be involved in transport of Zn within and between different parts of rice plant, and their expression varied with the different Zn conditions (Ramesh et al. 2003; Ishimaru et al. 2007, 2011). The *OsZIP1* gene was up regulated under Zn deficient conditions, while *OsZIP3* was up regulated both under controlled and Zn deficient conditions in rice (Ramesh et al. 2003). Over expression of *OsIRT* and *MxIRT* gene in rice resulted in increased Fe and Zn concentration in rice grains (Lee and An 2009; Tan et al. 2015). Similarly, *OsOZT1*, *OsVIT1* and *OsVIT2* are important vacuole metal transporters involved in Zn transport across the tonoplast and also help in Zn sequestration within the cell (Lan et al. 2013; Zhang et al. 2012). While, *OsYSL* family proteins play an important role in phloem transport and long distance transport of metals (Inoue et al. 2009; Aoyama et al. 2009; Lee et al. 2009; Ishimaru et al. 2010; Sasaki et al. 2011; Kakei et al. 2012). The *OsYSL2* gene has increased the Fe content in rice by 4 folds (Ishimaru et al. 2010; Masuda et al. 2013). Over-expression of *OsHMA3* enhanced the uptake of Zn by up regulating the ZIP family genes in the roots (Sasaki et al. 2014). Whereas, *OsHMA2* gene was involved in loading of Zn to the developing tissues in rice (Yamaji et al. 2013). Several studies have shown that the over expression of *OsNAS* genes improved the grain Fe and Zn concentrations by several folds, *OsNAS2* and *OsNAS3* over expression showed increased accumulation of Fe and Zn (Lee et al. 2011; Johnson et al. 2011). *OsIRO2* increases Fe content in rice plants grown in calcareous soils (Ogo et al. 2011). The ferritin gene *OsFer2* over expressed in a basmati rice (Pusasugandh II) accumulated higher levels of Fe and Zn (Paul et al. 2012). Several transcription factors such as *OsNAC*, *NAM-B1*, *OsIDEF1*, *OsIDEF2* and *OsIRO2* also play an important role in up regulating the genes involved in metal homeostasis (Ogo et al. 2006, 2007, 2008; Waters et al. 2009; Banerjee et al. 2010; Ogo et al. 2011; Gande et al. 2014). In an expression analysis study with 25 metal-related genes revealed that nine genes such as *OsYSL6*, *OsYSL8*, *OsYSL14*, *OsNRAMP1*, *OsNRAMP7*, *OsNRAMP8*, *OsNAS1*, *OsFRO1* and *OsNAC5* were specifically over expressed in the flag leaves and showed significant correlations with Fe and/or Zn concentrations in the seeds (Sperotto et al. 2010). Similarly, transcriptome analysis of 25 metal homeostasis genes in different tissues of 12 rice genotypes showed expression of highest number of genes (24) in flag leaf, while genes such as *OsZIP4*, *OsZIP11*, *OsNRAMP5*, *OsNRAMP7*, *OsYSL2*, *OsYSL4*, *OsYSL6*, *OsYSL9*, *OsNAAT1, OsNAC*, *OsFER1*, *OsVIT1*, *OsFRO2*, *OsIRT1*, *OsFER2*, *OsZIP7*, *OsZIP8*, *OsZIP9*, *OsNRAMP4*, *OsNRAMP6* and *OsYSL12* were expressed in roots. Expression of *OsNAC*, *OsYSL2*, *OsYSL9*, *OsZIP4*, *OsVIT1*, *OsNAAT1* and *OsNRAMP7* genes in the flag leaf was highly correlated with the high grain Zn content (Banerjee and Chandel 2011). Zn deficiency tolerant line RIL46 was found to produce higher level of deoxy mugineic acids and low molecular weight organic acids compared to non- tolerant line IR74 under Zn deficient conditions (Widodo et al. 2010). In a another study with RILs of Madhkar x Swarna, *OsNAS* and *OsHMA* were over expressed in the leaves (Priya et al. 2015), in the same set of materials under Fe deficient conditions *NAS2*, *IRT2*, *DMAS1* and *YSL15* were expressed in shoot, while *NAS2*, *IRT1*, *IRT2*, *YSL2* and *ZIP8* in the roots (Agarwal et al. 2014). Similarly, Chadha-Mohanty et al. (2015) reported that *OsZIP5* and *OsFRO1* were up regulated in roots and flag leaf of high Zn rice lines. Thus, it is very clear that several genes and gene networks are involved in metal uptake, translocation, sequestration and loading, and their well coordinated action play a key role in metal homeostasis in the rice plants.

Several QTLs for grain Zn have been mapped using various rice germplasm resources such as rice land races, *indica*, *japonica*, *aus* accessions, and wild rice species, viz., *O. rufipogon* and *O. nivara* (Lu et al. 2008; Garcia-Oliveira et al. 2009; Norton et al. 2010; Swamy et al. 2011). Different mapping populations such as Recombinant Inbred Lines (RILs), Double Haploids (DH), Backcross Inbred Lines (BILs), and Introgression Lines (ILs) have been used in grain Zn QTL mapping studies (Stangoulis et al. 2007; Lu et al. 2008; Garcia-Oliveira et al. 2009; Norton et al. 2010; Zhang et al. 2011; Anuradha et al. 2012b). Details of the QTLs identified, mapping populations used, size of the population, marker intervals, phenotypic variance (PV) and additive effects are presented in Table 2. In all there were 26 QTLs reported from eight different studies. It is clearly evident that genes/QTLs for high grain Zn are distributed throughout the genome and also found to co-locate with QTLs for other mineral elements in the grain. The number of QTLs on each chromosome varied from 1 to 6. One QTL each on chromosomes 1, 3, 8, 9, 10 and 11, two QTLs each on chromosomes 2, 4 and 6, three QTLs on chromosome 5, five and six QTLs on chromosome 12 and 7 respectively. QTLs on chromosome 7 and 12 were found to be more consistent across the genetic backgrounds and environments. Sixteen QTLs had more than 10 % PV and it varied from 5.0 % (*qZn5-1*) to 35.0 % (*qZn7.2* and *qZn12*) (Anuradha et al. 2012b). The additive effect of the QTLs varied from 0.4 ppm (*qZn4, qZn6)* to 17.1 ppm (*qZn12.2*). QTLs such as *qZn1-1, qZn3.1 qZn7.1 qZn7.2, qZn7.3, qZn8-1, qZn12.1, qZn12.2, qSZn2* and *qSZn12* had PV of more than 10 % with an additive effect of more than 5 ppm. The consistently identified grain Zn QTLs on chromosomes 7, 11, 12 are good targets for MAS. Thus, it is possible to increase the grain Zn concentration by 10 to 15 ppm in the existing popular rice varieties by a well designed Maker Assisted QTL pyramiding program.

**Table 2:** Details of QTLs identified for grain Zn in different studies

SN	Parentage	Pupulation	Population Size	QTL	Flanking marker	R^2^ (%)	Additive effect	positive allele	References
1	Zhengshan 97 × Minghui 63	RIL	241	*qZn-5*	R3166-RG360	12.3	−2.3	Zhengshan 97	Lu et al. 2008
				*qZn-7*	RM234-R1789	5.3	−1.5	Zhengshan 97	
				*qZn-11*	C794-RG118	18.6	2.9	Minghui 63	
2	Teqing × *O rufipogon*	BIL	85	*qZn5-1*	RM1089	5.0	−2.2	TeQing	Garcia-Oliveira et al. 2009
				*qZn8-1*	RM152	19.0	5.0	*Oryza rufipogon*	
				*qZn12-1*	RM3331	9.0	6.9	*Oryza rufipogon*	
3	IR64 × Azucena	DH	129	*qZn1-1*	RM34–RM237	15.0	5.4	Azucena	Stangoulis et al. 2007
				*qZn12-1*	RM235–RM17	12.8	1.6	Azucena	
	ZYQ8 × JX17	DH	127	*qZn4*	CT206-G177	10.8	0.4	JX17	Zhang et al. 2011
				*qZn6*	RZ516-G30	12.3	0.4	JX17	
4	Madhukar × Swarna	RIL	168	*qZn3.1*	RM7–RM517	31.0	11.01	Madhukar	Anuradha et al. 2012b
				*qZn7.1*	RM234–RM248	35.0	13.3	Madhukar	
				*qZn7.2*	RM248–RM8007	35.0	13.3	Madhukar	
				*qZn7.3*	RM501–OsZip2	29.0	−11.4	Swarna	
				*qZn12.1*	RM17–RM260	35.0	−16.2	Swarna	
				*qZn12.2*	RM260–RM7102	34.0	−17.1	Swarna	
5	Bala × Azucena		158	*qZn7*	R1440	12.0	-	Azucena	Norton et al. 2010
6	Sasanishiki × Habataki	BIL	85	*qSZn2*	R418–C1221	16.7	−16.0	Habataki	Ishikawa et al. 2010
				*qSZn12*	R1709–C1069	21.3	15.9	Sasanishiki	
7	TeQing × Lemont	IL	123	*qZn2*	RM106	-	−0.8	Lemont	Zhang et al. 2014
				*qZn4*	RM317	-	−1.4	Lemont	
				*qZn5*	RM421	8.1	−0.5	Lemont	
				*qZn6*	RM435	-	−1.5	Lemont	
				*qZn7*	RM248	-	−0.9	Lemont	
				*qZn9*	RM3909	-	1.1	TeQing	
8	Lemont × TeQing	RIL	280	*qZn10*	RG241a	4.4	−0.5	Lemont	Zhang et al. 2014

Several gene specific markers such as *OsZIP1, OsZIP3, OsZIP4, OsZIP5, OsZIP8, OsZIP8a*, *OsYSL8, OsYSL9, OsFRO2A, OsNAS1*, *OsNAS2*, *OsNAS3, OsArd2*, *OsIRT1*, *OsIRT2* and *OsNRAMP1*, showed a very good association with grain Zn (Giraldo et al. 2008; Chandel et al. 2011; Anuradha et al. 2012b; Gande et al. 2014). Similarly, based on the expression analysis of 21 metal homeostasis genes in 12 diverse rice genotypes, 179 novel SNPs and 39 SSR markers were identified for grain Zn (Banerjee et al. 2010). Several SSR markers and grain Zn trait associations have also been identified in different populations and germplasm panel of rice (Hanamareddy et al. 2007; Susanto 2008; Zhang et al. 2013; Brara et al. 2015). All these tightly linked SNP and SSR markers can be used in MAS. However, there is no literature indicating the successful use of these markers in MAS for improving grain Zn in rice. So, before using these QTLs/genes in MAS further validation on a large panel of high Zn donor lines and Zn specific biparental mapping populations is essential. A QTL pyramiding approach with different combinations of these consistent major effect QTLs can be tried in MAS for high grain Zn. As some of these QTLs have large intervals, fine mapping, candidate gene identification, and development of gene specific markers may facilitate their use in MAS.

It is also interesting to note that there is a highly significant positive correlation between grain Fe and Zn concentration and it is evident by the co-location of QTLs for Fe and Zn in brown rice, and there is no such strong correlation between these two elements in polished rice (Sala et al. 2013). Grain Zn QTLs also found to co-locate with QTLs for other mineral elements such as Cu, Cr, Mg, Si and Se (Hanamareddy et al. 2007; Du et al. 2013). Introgression of such QTLs will help to enhance the levels of multiple beneficial mineral elements in rice grains.

The expression of QTLs for grain Zn may be consistent or may vary with the genetic background and environment. Garcia-Oliveira et al. (2009) identified two grain Zn QTLs such as *qZn5-1* and *qZn8-1* consistently over two years. Bekele et al. (2013) identified seven marker and Zn trait associations; five of these were consistently identified over two seasons, while two of them were evident only in one season. Du et al. (2013) evaluated a DH population in two different locations and identified three different QTLs in each location, indicating the environment specificity of QTLs. Based on selective genotyping, two loci on chromosome 3 and one locus on chromosome 4 were consistently identified for grain Zn in two populations derived from Chittimutyalu and Ranbir Basmati (Babu 2013). In a Genome-Wide Association (GWAS) mapping for grain Zn and other elements in a rice diversity panel of 421 accessions, representing five sub populations of rice, including *indica*, *tropical japonica*, *temperate japonica*, *aus*, and *aromatic*, evaluated over five locations identified significant SNPs at 22.26 Mb on chromosome 3, consistently associated with grain Zn over four locations, while another set of SNPs identified on chromosome 9 were associated with grain Zn only in the *indica* and *aus* sub-populations (Norton et al. 2014). All these results clearly indicates significant G x E for grain Zn accumulation. Thus, choosing QTLs or their combinations based on the genetic background of recipient varieties and intended target environment is important before embarking on MAS (Swamy et al. 2012, 2013a, 2014).

In addition to main effect QTLs, several epistatic QTLs were also identified for grain Zn. Lu et al. (2008) reported six epistatic loci with additive and additive interactions for grain Zn, which accounted for 50.2 % of the total heritability of the trait. Norton et al. (2010) also reported `epistasis for grain Zn, which accounted for 20 % of the PV between chromosome 7 (G338-C39) and 9 (G1085-AB0905) with a LOD of 4.5, indicating strong genetic control involving multiple QTLs/genes. Apart from identifying QTLs for grain Zn, several QTLs have also been identified for Zn in other plant parts. Norton et al. (2010) identified a QTL with a PV of 12 % for Zn in leaf and four QTL were identified for grain Zn with a PV of 11 to 15 %. There was a little correlation between leaf Zn and grain Zn concentration, also the QTLs for Zn concentration in leaf and grain Zn are found on different chromosomal locations indicating different mechanisms responsible for Zn accumulation in vegetative and reproductive tissues (Norton et al. 2010; Nagarathna et al. 2010). In an IR64 × Jalamagna population, six QTLs were identified for Zn concentration in root and shoot; all were minor alleles and also showed epistatic effects. Some of them co-located with QTLs identified for grain Zn, Zn toxicity tolerance, and Zn deficiency tolerance (Dong et al. 2006; Wissuwa et al. 2006). Similarly, in a Sasanishiki × Habataki BIL population, two QTLs *qSZn2* and *qSZn12* with a PV of 16 % and 21 % were identified for straw Zn concentration (Ishikawa et al. 2010). All these recent advances in understanding of molecular basis of grain Fe and Zn should be used in increasing the efficiency of Zn biofortifcation of rice.

### Agronomic interventions to enhance grain Zn

An adequate amount of plant available Zn in the soil is essential for Zn biofortified rice genotypes to accumulate Zn in grains. Most of the rice growing area is Zn deficit and also Zn availability in irrigated rice ecosystems is very low due to formation of less soluble Zn complexes under anaerobic conditions. An estimation of soil Zn status and application of Zn fertilizer to Zn deficit soil is important for Zn biofortification. Agronomic Zn biofortification through Zn fertilizer application is a complementary approach to increase grain Zn concentration in new elite rice genotypes to ensure adequate root Zn uptake and transport to the grains during reproductive growth stage (Shivay et al. 2008; Phattarakul et al. 2012). The kind of Zn fertilizer applied, timing, and method of application is crucial for enhancing grain Zn. Application of Zn fertilizer to Zn sufficient soil has shown inconsistent results and most of the Zn was found to accumulate in vegetative tissues rather in grains, however in Zn deficit soil, Zn fertilizers improved grain Zn concentrations of rice (Wissuwa et al. 2008; Johnson-Beebout et al. 2009). Further, the response to Zn fertilizer has been shown to differ across rice genotypes and soil conditions (White and Broadley 2011; Jiang et al. 2008). Foliar application of Zn fertilizers has shown better results than soil application for increasing grain Zn concentration, but the magnitude of this increase is not consistent across genotypes (Wei et al. 2012; Mabesa et al. 2013). The effect of Nitrogen fertilizer application on rice grain Zn concentration has also shown inconsistent results, but in general, increasing Nitrogen application negatively influences grain Zn (Moraghan et al. 1999; Zhang et al. 2008; Chandel et al. 2010; Gao et al. 2010; Kutman et al. 2010; Shi et al. 2010). The application of gypsum is useful to remove bicarbonate from the soil solution, and can be highly beneficial for lowering the soil pH thereby increasing the availability of micronutrients including Zn in alkali and sodic soils (Rengel et al. 1999).

Bio-fertilizers such as *Mycorrhiza*, *Azolla, Rhizobacter*, *Azospirillum*, Zn-solubilizing bacteria, and organic manures enhance the levels of bioavailable Zn under flooded conditions and also shown to increase the Zn in rice grains (Tariq et al. 2007; Singh and Prasad 2014; Vaid et al. 2014; Wang et al. 2014; Subedi and Shrestha 2015), but their application on large scale biofortification programs needs to be carefully studied.

Water and crop residue management also significantly influence the Zn availability in continuous flooded soils. Zn forms less soluble Zn complexes under anaerobic conditions while in aerobic soils, free Zn is available to the plant (Johnson-Beebout et al. 2009; Impa and Johnson-Beebout 2012). Alternate wetting and drying (AWD) technology, which has been advocated for rice cultivation as a water saving technology also found to increase the Zn in grains. A combination of suitable rice genotype, AWD water management and ZnSO_4_ fertilization increased Zn accumulation and bioavailability in rice grains (Wang et al. 2014). A high Zn rice genotype Maligaya Special (MS13) showed consistent accumulation of Zn both under flooded and aerobic conditions (Nemeño et al. 2010). Therefore, development of high Zn rice genotypes with better accumulation of Zn across the water regimes and agronomic management without any yield penalty is highly desirable for efficient rice Zn biofortification. Crop rotation and intercropping of rice with other cereals and legumes could improve the Zn availability (Rengel et al. 1999). Rice-wheat crop rotation, application of farmyard manure and green manure can maintain the available fraction of soil micronutrients such as Fe, Zn, Cu, and Mn (Karlen et al. 1994; Kumar and Yadav 1995).

Agronomic management of the rice crop during breeding for high Zn rice is one of the most important considerations since Zn status in the soil and water management affects the availability of Zn and finally influences the grain Zn. All the early and advanced generation breeding materials should be evaluated in locations where soil Zn is homogeneous and not limiting, and water management is carefully controlled throughout the cropping cycle. This may be achieved by selecting plots which are naturally homogenous or applying a high rate of Zn fertilizer to Zn deficit plot to homogenize the area. The latter may be done by planting a systematic check cultivar in a given area and developing maps using geo-statistics that show variability for Zn grain concentration. It is important to maintain an adequate amount of available Zn in the soil during the crop growth period and testing the performance of high Zn rice lines over a wide range of environments with different levels of Zn is essential before being released as varieties as most parts of the rice growing areas suffer Zn deficiency. A combination of best agronomic management practices and selection of appropriate rice genotypes is essential for successful rice biofortification.

### Breeding interventions to enhance grain Zn

The genetic biofortification strategy uses plant breeding techniques to produce staple food crops with higher micronutrient levels (HarvestPlus 2014). It offers a sustainable solution to malnutrition problems by exploring natural genetic variation to develop mineral-dense crop varieties (Bouis 2003; Pfeiffer and McClafferty 2008). There is a wide variation for grain Zn in the rice germplasm and it is possible to breed for high Zn rice by exploiting high-Zn donors.

#### Zn target to be achieved in rice by breeding

The rice varieties commonly grown by farmers have relatively low levels of Zn (<12–14 mg kg ^−1^) in polished rice and cannot meet the daily dietary requirement of Zn. HarvestPlus, which has a specific focus on crop biofortification, has determined a target for the Zn level to be achieved in rice. Based on the nutrient needs, daily food intake, retention and bioavailability analyses of people suffering from Zn deficiency, the Zn breeding target in rice was raised from the previous target level of 24 mg kg ^−1^ of Zn to a new target of 28 mg kg ^−1^ (HarvestPlus 2014). The new value is based on the daily requirement of Zn for women. Given the 422 g of daily average rice consumption, with a lower Zn absorption of 20 %, and with a retention of 90 %, a concentration of 28 mg kg ^−1^ of Zn in the parboiled and milled rice would be enough to attain the Estimated Average Zn Requirement (EAR) of 25 %, which is sufficient to overcome most of the severe Zn deficiency problems (HarvestPlus 2014).

#### High throughput phenotyping for grain Zn

Precision phenotyping of grain Zn concentration is vital for breeding high Zn rice variety. Since breeding programs handle huge amount of materials, fast, accurate, and inexpensive methods of phenotyping grain Zn are essential for making timely and effective selection decisions when advancing the material. Seed sampling, hulling, and milling procedures without any metal contaminations have already been standardized for rice (Stangoulis and Sison 2008). Traditionally, Atomic Absorption Spectrometry (AAS) and Inductively Coupled Plasma-Optical Emission Spectrometry (ICP-OES) are being used in elemental analysis (Zarcinas et al. 1987), while these methods are highly accurate, they require expensive equipment, highly trained analysts, contamination free reagents, and extensive sample preparation (Velu et al. 2013). Alternatively, colorimetric approaches have been developed for Zn and Fe analysis in different cereal crops; however, these approaches are only semi-quantitative and laborious when applied in large scale (Prom-u-thai et al. 2003; Ozturk et al. 2006; Choi et al. 2007; Velu et al. 2008). X-Ray Fluorescence (XRF) Spectrometry is very useful in non-destructive determination of relative Zn and Fe concentration in rice breeding lines to discard low Zn lines, and the resulting high Zn lines selected on the basis of XRF can be validated with ICP (Paltridge et al. 2012). Most of the biofortification programs are using XRF for metal analysis.

The Synchrotron based X-ray micro fluorescence imaging and isotope discrimination techniques are helpful in understanding the pathways of metal uptake, translocation and retranslocation, portioning and distributions among different tissues and organs. Takahashi et al. (2009) and Lu et al. (2013) characterized the dynamic changes in the pattern and distribution of different metals in germinating rice seedlings using X-ray imaging and concluded that metals have different patterns and preferences in their movement and accumulations. Arnold et al. (2015) used Isotope discrimination to study the Fe and Zn uptake and translocation in rice grown under different environmental conditions and results showed that different isotope fractionation for different metals in different organs/tissues and in different environments indicating different mechanisms involved in Fe and Zn homeostasis. This novel Isotope fractionation technique is highly useful in better understating the physiological mechanisms, and the genotype and environment interactions involved in the Zn accumulation in grains.

#### Identification of high Zn rice germplasm

Selection of donors with high grain Zn in polished rice, acceptable yield potential and other desirable traits with minimal linkage drag and without any crossing barriers is an essential foundation for a successful high Zn breeding program. There is abundant genetic variation for the grain Zn concentration in both brown and polished grains in the rice germplasm. Rice wild relatives, landraces, *aus* and aromatic accessions, deep water rice and colored rice are the best sources of high grain Zn. Wild species of rice such as *O. nivara, O. rufipogon, O. latifolia, O. officinalis,* and *O. granulata* also contain high amounts of Zn, around 2–3 fold higher than in the cultivated rice, with Zn concentration varying from 37 mg/kg to 55 mg/kg in non-polished grains (Cheng et al. 2005; Banerjee et al. 2010; Anuradha et al. 2012a). Aromatic rice has also shown high Zn compared to non-aromatic rice (Gregorio 2002).

#### Breeding strategies for developing high Zn rice

**Fig. 1:**
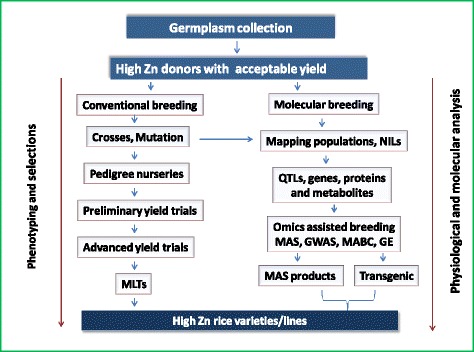
Breeding strategy for developing high Zn rice varieties

Genotypic variation for grain Zn concentration in rice can be exploited through breeding. For the past few years, breeding efforts to increase grain micronutrients have resulted in the development of biofortified crops including rice (HarvestPlus 2014). Since the genetic basis of grain Zn is complex with the involvement of multiple small effect genes/QTLs and significantly influenced by the environment, the choice of appropriate breeding methods, crossing programs, individual plant selections and field evaluation processes are critical for the successful development of high-Zn rice. Previously, high-Zn donors have been crossed with popular high-yielding but low-Zn rice varieties and selection was carried out for agronomic traits in the segregating generations, with final fixed lines tested for grain Zn and yield in replicated large scale plots. This method was time consuming and resulted in modest increase in the Zn concentration, while the lines developed had moderate yield potential. However, a modified breeding program using high-Zn donors with acceptable yield potential crossed with popular high yielding, highly adapted, but low-Zn rice varieties, coupled with Zn testing in early segregating lines from the F_4_ generation onwards along with the selections for acceptable agronomic traits, can hasten the process of high-Zn variety development and simultaneous maintenance of yield potential (Fig. 1). Multiple crosses involving several donors and recipient parents such as three-way, four-way crosses etc., reciprocal crosses with the donor parent, high Zn × high Zn crosses involving advanced Zn lines will enhance the Zn levels and yield potential. Multi-parent Advanced Generation Inter-Cross (MAGIC) is also an attractive method for pooling the genes for high Zn, and at IRRI several MAGIC populations such as MAGIC-*indica*, MAGIC- *japonica* and MAGIC-global (utilizing crosses between *indica* and *japonica* MAGIC lines) have been developed (Bandillo et al. 2013) and these are a good resource for selecting high Zn lines and also provides an opportunity to select transgressive segregants for high Zn.

Exploitation of heterosis of grain Zn and yield potential is also an important approach for developing high Zn rice hybrids. Reports have shown that there is very good heterosis for grain Zn in rice (Nagesh et al. 2012). Wild relatives of rice such as *O. nivara*, *O. rufipogon*, *O. barthii*, and *O. longistminata*, and African cultivated rice *O. glaberrima* are found to have higher level of Zn in the grains and these are a potential source of high Zn donors (Garcia-Oliveira et al. 2009; Sarla et al. 2012). Advanced backcross breeding method can be used to exploit these wild resources to combine high Zn and high yield potential, and this will also help to broaden the genetic base of the popular rice varieties. Mutation breeding is also gaining importance as a strategy to improve Zn concentration in rice. Physical and chemical mutagens have been used in mutation breeding and mutants with high Zn have been identified. A number of IR64 mutants produced by the treatment with Sodium azide were reported to have high Zn (Jeng et al. 2012). Three IR64 mutant lines viz; M-IR-180, M-IR-49, and M-IR-175 had more than 26 mg kg ^−1^ Zn in polished rice as against 16 mg kg ^−1^ in IR64. These mutants can be used as a donor in Zn breeding programs and are also a valuable resource for understanding the genetic mechanism involved in the expression of high Zn phenotype. There are reports indicating high correlations between Zn deficiency tolerance and high grain Zn in rice, so the selection of segregating recombinant inbred lines or mutants under Zn deficiency conditions followed by yield evaluation under normal conditions may also be a useful approach to improve the Zn concentration in rice.

Marker assisted breeding for high Zn rice using major effect grain Zn QTLs is also a more faster and precise approach. Several major effect grain Zn QTLs with a high PV (>10 %) and also gene-specific markers for grain Zn have been reported in rice, but use of these markers to assist breeding efforts to improve Zn concentration in rice has not been reported. There is a huge potential to use these markers in marker assisted breeding to improve grain Zn concentration in rice. Since there are many QTLs/ genes responsible for grain Zn concentration located on different chromosomes, QTL pyramiding, Marker Assisted Recurrent Selection (MARS) and Genomics Assisted Breeding approaches are worth trying to develop high-Zn rice.

SNPs are becoming markers of choice for many breeding programs. Several diverse SNP chips such as 1536 SNPs diversity panel, 44 K, 50 K, 1 M SNP chips are available for rice. The cheaper, faster and high throughput SNP assays made it possible the routine use of markers in the breeding programs (McCouch et al. 2010; Swamy and Kumar 2013b; Singh et al. 2015). The recent advances in sequencing technologies such as Next Generation/Second Generation Sequencing (NGS/SGS) and Third Generation Sequencing technologies (TGS) have revolutionized the breeding unprecedently (Varshney et al. 2009). Sequencing and resequencing of several thousands of accessions and breeding lines for use in sequence based mapping, genome wide association mapping, genome wide predictions and genomic selections have become possible with the help of these technologies (Deschamps et al. 2012). In rice, 3000 accessions have been sequenced and efforts are ongoing to sequence 10,000 accessions (Li et al. 2014). More than 20 M SNPs have been identified from 3 K panel (Alexandrov et al. 2015). High quality and high throughput sequencing coupled with Rapid Generation Advance (RGA) and high throughput phenotyping can hasten the breeding process especially for complex traits such as grain micronutrients. Genome Wide Association Studies (GWAS) and Genomic Selections (GS) approach have not been explored much for improving grain micronutrients but they hold great promise for improving the grain Zn concentration of several popular rice varieties and highly useful in main streaming of the Zn breeding.

While breeding for high Zn rice, special attention should be given to the amount of anti-nutrients as they significantly influence Zn bioavailability. Phytate is the major anti-nutrient inhibiting the quantity of Zn absorbed. In rice, Zn is preferentially stored together with phytate, which is a strong chelator of divalent cations (Bohn et al. 2008; Hambidge et al. 2010; Petry et al. 2012). Hence, selections should be made for low phytate content. The use of high Zn donors with low phytate, selection of segregating lines and advanced fixed lines with low phytate, and integrating phytate phenotyping along with grain Zn in the breeding program will help in developing high Zn lines with low phytate content. Recently by mutation breeding, several mutants with low phytate content have been developed and are good resources as low-phytate donors in breeding programs (Liu et al. 2007).

### Current status of breeding for high Zn rice

Zn biofortified rice has a huge potential in combating malnutrition in rice consuming poor countries of Asia, Africa and Latin America. HarvestPlus in collaboration with International Rice Research Institute (IRRI) and International Center for Tropical Agriculture (CIAT) and National Agricultural Research and Extension Systems (NARES) partners in several countries are carrying out programs to develop high Zn rice varieties (Bouis et al. 2013). International Rice Research Institute (IRRI) is also making efforts to include high grain micronutrient trait as an integral part of all the mainstream breeding projects. The major target countries of the rice Zn biofortification program are India, Bangladesh, Indonesia and the Philippines. The biofortification breeding team at IRRI has identified several high Zn rice germplasm as donors, early generation and also advanced high Zn material in the background of popular rice varieties such as IR64, Swarna, Swarna Sub1, NSICRc222, PSBRc82, BR28, BR29, BR11, and Ciherang has been produced and shared with national partners. Overall, IRRI is coordinating the breeding activities of the country partners and also encouraging them to generate high Zn material in the genetic backgrounds of locally adopted popular rice varieties using the high Zn donors supplied by IRRI (Swamy et al. 2015). The first installment of high Zn materials with an additional 6–8 mg kg ^−1^ of Zn (18–20 mg kg ^−1^ as against 12–14 mg kg ^−1^ Zn of popular varieties) are ready for release in the partner countries, and a second cohort of high Zn lines with additional 8–10 mg kg ^−1^ of Zn are in the development pipeline. Two high Zn rice varieties BRRI dhan 62 and BRRI dhan 64 have been released for cultivation during the Aman and Boro seasons in Bangladesh. These two varieties have 19 mg kg ^−1^ and 25 mg kg ^−1^ of Zn with a yield potential of 4.2 t/ha and 6 t/ha respectively (HarvestPlus 2014), and also there are many high Zn lines in advanced stages of evaluation for varietal release in Bangladesh. IRRI has also shared with them early generation material combining high Zn and submergence tolerance, for further evaluation and selection in Bangladesh environments.

In the Philippines, the high Zn breeding materials shared by IRRI are in the Pre-National Cooperative Test (Pre-NCT) and National Cooperative Test (NCT) for release. There are many high Zn lines under evaluation in research station trials within the Philippines Rice Research Institute (Inabangan-Asilo et al. 2015). In Indonesia and India, high Zn rice lines are in the advanced stages of evaluation in the multi-location testing and in station trials. These first and second sets of high Zn lines have 18–22 mg kg ^−1^ of Zn with acceptable yield potential, grain quality and agronomic traits (Swamy et al. 2015). These lines can meet 16–20 % of the estimated average requirement of Zn, which is sufficient to overcome severe health problems caused by Zn deficiency. In the coming years we are hopeful of releasing several high Zn rice lines in the target countries and seeing them deployed on a large scale. The initial success of high Zn rice and high Zn cultivars of other crops which have been developed and released has provided further impetus to expand the program to other poor countries of Asia.

## Conclusions

Biofortification of rice with Zn is a cost-effective and sustainable solution to mitigate Zn deficiency problems in the rice consuming malnourished Asian populations. There is a significant genetic variation for grain Zn in rice germplasm resources which can be exploited by breeding to develop high Zn rice varieties. The recent advances in understanding the physiological, genetic and molecular basis of Zn uptake, Zn transport and loading into grains has allowed effective breeding for increased Zn, however the physiological barriers for loading Zn into grains are still a significant obstacle for attaining the targeted level of Zn. A complete understanding of Zn allocation, reallocation, and remobilization within and between vegetative and reproductive tissues is lacking. Agronomic interventions to improve the grain Zn have inconsistent results, but a combination of agronomic and genetic interventions is likely to prove a more effective approach. Several major effect and consistent QTLs for grain Zn have been identified; there is great potential to use them in MAS. Two high Zn rice lines have been released in Bangladesh, and several high Zn lines are in the advanced stages of evaluation for release in other partner countries.

## Additional file

Additional file 1: Table S1. List of Zinc homeostasis genes and their putative functions Chen et al. 2008; Dräger et al. 2004; Gross et al. 2003; Grotz & Guerinot 2006; Kobayashi et al. 2003; Kobayashi et al. 2005; Koike et al. 2004; Lee et al. 2010a; Lee et al. 2010b; Ricachenevsky et al. 2011; Sasaki et al. 2012; Stein et al. 2009; Takahashi et al. 2012; Vasconcelos et al. 2008; Yang et al. 2007; Yang et al. 2009a; Yang et al. 2009b; Yokosho et al. 2009; Yuan et al. 2012. (DOC 44 kb)

## Abbreviations

AAS: Atomic absorption spectrometry
ARD: Acireductone dioxygenase
BIL: Backcross inbred lines
CIAT: International Center for Tropical Agriculture
CV: Co-efficient of variation
DH: Doubled haploid
DMAS: Deoxy mugineic acid synthase
EAR: Estimated average requirement
FER: Ferritin
FRDL: Ferric reductase defective like
FRO: Ferrus reductase-oxidase
G x E: Genotype and environment interaction
GE: Genetic engineering
GS: Genomic selection
GWAS: Genome wide association study
HMA: Heavy metal associated proteins
ICP-OES: Inductive coupled plasma-optical emission spectrometry
IDEF: Iron deficiency-responsive cis-acting element binding factors
IL: Introgression lines
IRO: Iron regulated transcription factor
IRT: Iron regulated transporter
MABC: Marker assisted back cross
MAGIC: Multi-parent advance generation inter cross
MARS: Marker assisted recurrent selection
MAS: Marker assisted selection
MS13: Maligaya special 13
MT: Metallothionein protein
MTP: Metal transport protein
NAAT: Nicotianamine amino transferase
NAC: NAM, ATAF, CUC2
NAM: No Apical meristem
NARES: National Agricultural Research and Extension Systems
NAS: Nicotianamine synthase
NCT: National co-operative test
NGS: Next generation sequencing
NIL: Near isogenic lines
NRAMP: Natural resistance-associated-macrophage protein
OPT: Oligo peptide transport proteins
OZT: *Oryza sativa* zinc transport protein
PreNCT: Pre national co-operative test
PV: Phenotypic variance
QTL: Qunatitative trait loci
RGA: Rapid generation advance
RIL: Recombinant inbred lines
SAMS: S-adenosyl methionine synthase
SGS: Second generation sequencing
SNP: Single nucleotide polymorphism
SSR: Simple sequence repeats
TGS: Third generation sequencing
TOM: Transport of mugineic acid
VIT: Vacuole iron transporter
XRF: X-Ray fluorescence
YSL: Yellow stripe1-like
ZIF: Zinc induce facilitator like
ZIP: ZRT, IRT like Protein

## References

[CR1] Agarwal S, Venkata TVGN, Kotla A, Mangrauthia SK, Neelamraju S (2014). Expression patterns of QTL based and other candidate genes in Madhukar x Swarna RILS with contrasting levels of iron and zinc in unpolished rice grains. Gene.

[CR2] Alexandrov N, Tai S, Wang W, Mansueto L, Palis K, Fuentes RR, Ulat VJ, Chebotarov D, Zhang G, Li Z, Mauleon R, Hamilton RS, McNally KL (2015). SNP-Seek database of SNPs derived from 3000 rice genomes. Nucleic Acid Res.

[CR3] Anandan A, Rajiv G, Eswaran R, Prakash M (2011). Genotypic variation and relationships between quality traits and trace elements in traditional and improved rice (*Oryza sativa* L) genotypes. J Food Sci.

[CR4] Anuradha K, Agarwal S, Batchu AK, Babu AP, Swamy BPM, Longva T, Sarla N (2012a) Evaluating rice germplasm for iron and zinc concentration in brown rice and seed dimensions. J Geophys Res 4:19–25

[CR5] Anuradha K, Agarwal S, Rao VY, Rao KV, Viraktamath BC, Sarla N (2012b) Mapping QTLs and candidate genes for iron and zinc concentrations in unpolished rice of Madhukar × Swarna RILs. Gene 508:233–240 10.1016/j.gene.2012.07.054 22964359

[CR6] Aoyama T, Kobayashi T, Takahashi M, Nagasaka S, Usuda K, Kakei Y, Ishimaru Y, Nakanishi H, Mori S, Nishizawa NK (2009). *OsYSL18* is a rice iron (III)-deoxymugineic acid transporter specifically expressed in reproductive organs and phloem of lamina joints. Plant Mol Biol.

[CR7] Arnold T, Markovic T, Kirk GJD, Schonbachler M, Rehkamper M, Zhao FJ, Weiss DJ (2015) Iron and zinc isotope fractionation during uptake and translocation in rice (*Oryza sativa*) grown in oxic and anoxic soils. Geoscience, http://dx.doi.org/10.1016/j.crte.2015.05.005

[CR8] Babu VR (2013). Importance and advantages of rice biofortification with iron and zinc. J SAT Agric Res.

[CR9] Babu VR, Shreya K, Dangi KS, Usharani G, Nagesh P (2012). Genetic variability studies for qualitative and quantitative traits in popular rice (*Oryza sativa* L) hybrids of India. Int J Scientific and Res Publ.

[CR10] Bandillo N, Raghava C, Muyco PA, Sevilla MAL, Lobina IT, Dilla-Ermita CJ, Tung CW, McCouch S, Thomson M, Mauleon R, Singh RK, Gregorio G, Redoña E, Leung H (2013). Multi-parent advanced generation inter-cross (MAGIC) populations in rice: progress and potential for genetics research and breeding. Rice.

[CR11] Banerjee S, Chandel G (2011). Understanding the role of metal homeostasis related candidate genes in Fe/Zn uptake, transport and redistribution in rice using semi-quantitative RT-PCR. J Plant Mol Biol Biotechnol.

[CR12] Banerjee S, Sharma DJ, Verulkar SB, Chandel G (2010). Use of in silico and semi quantitative RT-PCR approaches to develop nutrient rich rice (*Oryza sativa* L) India. J Biotechnol.

[CR13] Banu H, Jagadeesh BN (2014). Genetic analysis of skewness and kurtosis for yield and its parameters, total grain protein, macro and micro nutrients in F_7_ generation of rice (*Oryza Sativa* L). Environ. Ecol..

[CR14] Bashir K, Inoue H, Nagasaka S, Takahashi M, Nakanishi H, Mori S, Nishizawa NK (2006). Cloning and characterization of deoxymugineic acid synthase genes from graminaceous plants. J Biol Chem.

[CR15] Bekele DB, Naveen GK, Rakhi S, Shashidhar HE (2013). Genetic evaluation of recombinant inbred lines of rice (*Oryza sativa* L) for grain Zinc concentrations, yield related traits and identification of associated SSR markers. Pak J Biol Sci.

[CR16] Berti C, Faber M, Smuts CM (2014). Prevention and control of micronutrient deficiencies in developing countries: current perspectives. Nutr. Diet. Suppl..

[CR17] Bohn L, Meyer AS, Rasmussen SK (2008). Phytate: impact on environment and human nutrition. A challenge for molecular breeding. J Zhejiang Univ Sci B.

[CR18] Bouis HE (2003). Micronutrient fortification of plants through plant breeding: can it improve utrition in man at low cost?. Precede Nutr Soc.

[CR19] Bouis HE, Welch RM (2010). Biofortification—A sustainable agricultural strategy for reducing micronutrient malnutrition in the global South. Crop Sci.

[CR20] Bouis H, Low J, McEwan M, Tanumihardjo S (2013) Biofortification: evidence and lessons learned linking agriculture and nutrition., http://www.fao.org/fileadmin/user_upload/agn/pdf/Biofortification_paper.pdf

[CR21] Brara B, Jaina RK, Jain S (2015). Correlation of molecular marker allele size with physio-morphological and micronutrient (Zn, Fe) traits among rice genotypes. Int J Current Sci.

[CR22] Cakmak I (2008). Enrichment of cereal grains with zinc: agronomic or genetic biofortification?. Plant Soil.

[CR23] Chadha-Mohanty P, Rey J, Francisco PB, Virk PS, Hossain MA, Swamy BPM (2015). Expression analysis of high zinc rice breeding lines using known homeostasis genes involved in iron and zinc acquisition and translocation.

[CR24] Chandel G, Banerjee S, See S, Meena R, Sharma DJ, Verulkar SB (2010). Effects of different nitrogen fertilizer levels and native soil properties on rice grain Fe, Zn and protein contents. Rice Sci.

[CR25] Chandel G, Samuel P, Dubey M, Meena R (2011). In silico expression analysis of QTL specific candidate genes for grain micronutrient (Fe/Zn) content using ESTs and MPSS signature analysis in rice (*Oryza sativa* L). J Plant Genet Transgenics.

[CR26] Chen WR, Feng Y, Chao YE (2008). Genomic analysis and expression pattern of OsZIP1, OsZIP3, and OsZIP4 in two rice (Oryza sativa L.) genotypes with different zinc efficiency. Russ J Plant Physiol.

[CR27] Cheng ZQ, Huang XQ, Zhang YZ, Qian J (2005). Diversity in the content of some nutritional components in husked seeds of three wild rice species and rice varieties in Yunnan Province of China. J Integr Plant Bio.

[CR28] Choi EY, Graham RD, Stangoulis JCR (2007). Rapid semi-quantitative screening methods for determination of iron and zinc in grains and staple foods. J. Food Compos. Anal..

[CR29] Department of Health (1991). Dietary reference values for food energy and nutrients for the United Kingdom, Report of the Panel on Dietary Reference Values of the Committee on Medical Aspects of Food Policy. Rep Health Soc.

[CR30] Deschamps S, Llaca V, May GD (2012). Genotyping-by-Sequencing in Plants. Biology.

[CR31] Dong Y, Ogawa T, Lin D, Koh HJ, Kamiunten H, Matsuo M, Cheng S (2006). Molecular mapping of quantitative trait loci for zinc toxicity tolerance in rice seedling (*Oryza sativa* L). Field Crop Res.

[CR32] Dräger DB, Fonrouge AD, Krach C, Chardonnens AN, Meyer RC, Laprade PS, Krämer U (2004). Two genes encoding *Arabidopsis halleri* MTP1 metal transport proteins co-segregate with zinc tolerance and account for high *MTP1* transcript levels. Plant J.

[CR33] Du J, Zeng D, Wang B, Qian Q, Zheng S, Ling HQ (2013). Environmental effects on mineral accumulation in rice grains and identification of ecological specific QTLs. Environ Geochem Health.

[CR34] Gande NK, Soman R, Kundur PJ, Ambati R, Bekele DB, Shashidhar HE (2013). Evaluation of recombinant inbred lines of rice (*Oryza sativa* L.) for grain zinc content, yield related traits and identification of transgressant lines grown under aerobic conditions. Asian J. Exp. Biol. Sci.

[CR35] Gande NK, Kundur PJ, Soman R, Ambati R, Ashwathanarayana R, Bekele BD, Shashidhar HE (2014). Identification of putative candidate gene markers for grain zinc content using recombinant inbred lines (RIL) population of IRRI38 × Jeerigesanna. Afr J Biotechnol.

[CR36] Gangashetty P, Salimath PM, Hanamaratti NG (2013). Association analysis in geneticallydiverse non-basmati local aromatic genotypes of rice (*Oryza sativa* L). Mol Plant Breed..

[CR37] Gao X, Hoffland E, Stomph T, Grant CA, Zou C, Zhang F (2006). Improving zinc bioavailability in transition from flooded to aerobic rice- A review. Agron Sustain Dev.

[CR38] Gao X, Brown KR, Racz GJ, Grant CA (2010). Concentration of cadmium in durum wheat as affected by time source and placement of nitrogen fertilization under reduced and conventional-tillage management. Plant Soil.

[CR39] Garcia-Oliveira AL, Tan L, Fu Y, Sun C (2009). Genetic identification of quantitative trait loci for contents of mineral nutrients in rice grain. J Integr Plant Biol.

[CR40] Giraldo OX, Quintero C, Plata G, Rodriquez F, Borrero J, Martinez CP, Tohme J (2008) Identification of SNP markers for biofortification in rice., CIAT Cali Colombia, http://ciat-library.ciat.cgiar.org/articulos_ciat/poster05_exhibit08.pdf

[CR41] Gregorio GB (2002). Progress in breeding for trace minerals in staple crops. J Nutr.

[CR42] Gross J, Stein RJ, Fett-Neto G, Fett JP (2003). Iron homeostasis related genes in rice. Genet. Mol. Biol..

[CR43] Grotz N, Guerinot ML (2006). Molecular aspects of Cu, Fe and Zn homeostasis in plants. Biochim Biophys Acta.

[CR44] Hacisalihoglu G, Kochian LV (2003). How do some plants tolerate low levels of soil zinc? Mechanisms of zinc efficiency in crop plants. New Phytol.

[CR45] Hambidge KM, Miller LV, Westcott JE, Sheng X, Krebs NF (2010). Zinc bioavailability and homeostasis. Am J Clin Nutr.

[CR46] Hanamareddy B, Bhargavi M, Ravindra S, Ramakrishna P, Shailaja H (2007). Identification of QTL associated with silicon and zinc content in rice (*Oryza sativa* L) and their role in blast disease resistance. Indian J. Genet. Plant Breed..

[CR47] Hansen TH, Laursen KH, Persson DP, Pedas P, Husted S, Jan K, Schjoerring JK (2009). Micro-scaled high-throughput digestion of plant tissue samples for multi-elemental analysis. Plant Methods.

[CR48] HarvestPlus (2014). Biofortification progress briefs.

[CR49] Hotz C, Brown KH (2004). Assessment of the risk of zinc deficiency in populations and options for its control. Food Nutr Bull.

[CR50] Impa SM, Johnson-Beebout SE (2012). Mitigating zinc deficiency and achieving high grain Zn in rice through integration of soil chemistry and plant physiology research. Plant and Soil.

[CR51] Impa SM, Morete MJ, Ismail AM, Schulin R, Johnson-Beebout SE (2013a) Zn uptake translocation and grain Zn loading in rice (*Oryza sati*va L) genotypes selected for Zn-deficiency tolerance and high grain Zn. J Exp Bot 64:2739–2751 10.1093/jxb/ert118 PMC3697949 23698631

[CR52] Impa SM, Gramlich AS, Tandy S, Schulin R, Frossard E, Johnson-Beebout SE (2013b) Internal Zn allocation influences Zn deficiency tolerance and grain Zn loading in rice (*Oryza sativa* L). Front Plant Sci 4:534 10.3389/fpls.2013.00534 PMC3871718 24400015

[CR53] Inabangan-Asilo MA, Amery Amparado A, Manito C, Tesoro F, Swamy BPM (2015). Development of high grain zinc rice varieties to alleviate zinc malnutrition.

[CR54] Inoue H, Higuchi K, Takahashi M, Nakanishi H, Mori S, Nishizawa NK (2003). Three rice nicotianamine synthase genes, *OsNAS1*, *OsNAS2*, and *OsNAS3* are expressed in cells involved in long-distance transport of iron and differentially regulated by iron. Plant J.

[CR55] Inoue H, Takahashi M, Kobayashi T, Suzuki M, Nakanishi H, Mori S, Nishizawa NK (2008). Identification and localisation of the rice nicotianamine aminotransferase gene *OsNAAT1* expression suggests the site of phytosiderophore synthesis in rice. Plant Mol Biol.

[CR56] Inoue H, Kobayashi T, Nozoye T, Takahashi M, Kakei Y, Suzuki K (2009). Rice *OsYSL15* is an iron-regulated iron(III)-deoxymugineic acid transporter expressed in the roots and is essential for iron uptake in early growth of the seedlings. J Biol Chem.

[CR57] Institute of Medicine Food and Nutrition Board (IMFNB) (2001). Dietary ReferenceIntakes for Vitamin A Vitamin K Arsenic Boron Chromium Copper Iodine Iron Manganese Molybdenum Nickel Silicon Vanadium and Zinc.

[CR58] Ishikawa S, Abe T, Kuramata M, Yamaguchi M, Ando T, Yamamoto T, Yano M (2010). A quantitative trait locus for increasing cadmium-specific concentration in rice grain is located on short arm of chromosome 7. J Exp Bot.

[CR59] Ishimaru Y, Suzuki M, Kobayashi T, Takahashi M, Nakanishi H, Mori S, Nishizawa NK (2005). *OsZIP4*, a novel zinc-regulated zinc transporter in rice. J Exp Bot.

[CR60] Ishimaru Y, Masuda H, Suzuki M, Bashir K, Takahashi M, Nakanishi H, Mori S, Nishizawa NK (2007). Over expression of the *OsZIP4* zinc transporter confers disarrangement of zinc distribution in rice plants. J Exp Bot.

[CR61] Ishimaru Y, Masuda H, Bashir K, Inoue H, Tsukamoto T, Takahashi M, Nakanishi H, Aoki N, Hirose T, Ohsugi R (2010). Rice metal-nicotianamine transporter, *OsYSL2* is required for the long-distance transport of iron and manganese. Plant J.

[CR62] Ishimaru Y, Bashir K, Nishizawa N (2011). Zn uptake and translocation in rice plants. Rice.

[CR63] Jeng TL, Lin YW, Wang CS, Sung JM (2012). Comparisons and selection of rice mutants with high iron and zinc contents in their polished grains that were mutated from the *indica* type cultivar IR64. J. Food Compos. Anal..

[CR64] Jiang SL, Wu JG, Feng Y, Yang XE, Shi CH (2007). Correlation analysis of mineral element contents and quality traits in milled rice (*Oryza sativa* L). J Agric Food Chem.

[CR65] Jiang W, Struik PC, VanKeulen H, Zhao M, Jin LN, Stomph TJ (2008). Does increased zinc uptake enhance grain zinc mass concentration in rice?. Ann Appl Biol.

[CR66] Johnson AAT, Kyriacou B, Callahan DL, Carruthers L, Stangoulis J (2011). Constitutive overexpression of the OsNAS gene family reveals single‐gene strategies for effective iron- and zinc-biofortification of rice endosperm. PLoS One.

[CR67] Johnson-Beebout SE, Lauren JG, Duxbury JM (2009). Immobilization of zinc fertilizer in flooded soils monitored by adapted DTPA soil test. Commun Soil Sci Plant Anal.

[CR68] Kakei Y, Ishimaru Y, Kobayashi T, Yamakawa T, Nakanshi H, Nishizawa NK (2012). *OsYSL16* plays a role in the allocation of iron. Plant Mol Biol.

[CR69] Karlen DL, Varvel GE, Bullock DG, Cruse RM (1994). Crop rotations for the 21st century. Adv Agron.

[CR70] Keith A, McCall KA, Huang C, Fierke CA (2006). Function and mechanism of zinc metallo enzymes. J Nutr.

[CR71] Kennedy G, Burlingame B, Nguyen VN (2002). Nutritional contribution of rice and impact of biotechnology and biodiversity in rice-consuming countries.

[CR72] Kobayashi T, Nakayama Y, Itai RN, Nakanishi H, Yoshihara T, Mori S, Nishizawa NK (2003). Identification of novel cis-acting elements, *IDE1* and *IDE2*, of the barley *IDS2* gene promoter conferring iron-deficiency-inducible, root-specific expression in heterogeneous tobacco plants. Plant J.

[CR73] Kobayashi T, Suzuki M, Inoue H, Itai RN, Takahashi M, Nakanishi H, Mori S, Naoko K, Nishizawa NK (2005). Expression of iron-acquisition-related genes in iron-deficient rice is coordinately induced by partially conserved iron-deficiency-responsive elements. J Exp Bot.

[CR74] Koike S, Inoue H, Mizuno D, Takahashi M, Nakanishi H, Mori S (2004). *OsYSL2* is a rice metal-nicotianamine transporter that is regulated by iron and expressed in the phloem. Plant J.

[CR75] Krishnan S, Dayanandan P (2003). Structural and histochemical studies on grain-filling in the caryopsis of rice (*Oryza sativa* L). J Bioscience.

[CR76] Kumar A, Yadav DS (1995). Use of organic manure and fertilizer in rice (*Oryza sativa*)-wheat (*Triticum aestivum*) cropping system for sustainability. Ind J Agric Sci.

[CR77] Kutman UB, Yildiz B, Ozturk L, Cakmak I (2010). Biofortification of durum wheat with zinc through soil and foliar applications of nitrogen. Cereal Chem.

[CR78] Lan H, Wang M, Zhang H, Wang Z, Bao Y, Wang Q, Huang J (2013). Characterization of a vacuolar zinc transporter *OZT1* in rice (*Oryza sativa* L.). Mol Biol Rep.

[CR79] Lee S, An G (2009). Over-expression of *OsIRT1* leads to increased iron and zinc accumulations in rice. Plant Cell Environ.

[CR80] Lee S, Chiecko JC, Kim SA, Walker EL, Lee Y, Guerinot ML, An G (2009). Disruption of *OsYSL15* leads to inefficiency in rice plants. Plant Physiol.

[CR81] Lee S, Jeong H, Kim S, Lee J, Guerinot M, An G (2010a) *OsZIP5* is a plasma membrane zinc transporter in rice. Plant Mol Biol 73:507–517 10.1007/s11103-010-9637-0 20419467

[CR82] Lee S, Kim S, Lee J, Guerinot M, An G (2010b) Zinc deficiency-inducible *OsZIP8* encodes a plasma membrane-localized zinc transporter in rice. Mol Cells 29:551–558 10.1007/s10059-010-0069-0 20496122

[CR83] Lee S, Persson DP, Hansen TH, Husted S, Schjoerring JK, Kim Y, Jeon US, Kim Y, Kakei Y, Masuda H, Nishizawa NK, An G (2011). Bio-available zinc in rice seeds is increased by activation tagging of *nictoianamine synthase*. Plant Biotechnol J.

[CR84] Li J, Wang J, Zeigler RS (2014). The 3,000 rice genomes project: new opportunities and challenges for future rice research. Gigascience.

[CR85] Liu QL, Xu XH, Ren XL, Fu HW, Wu DX, Shu QY (2007). Generation and characterization of low phytic acid germplasm in rice (*Oryza sativa* L). Theor Appl Genet.

[CR86] Lu K, Li L, Zheng X, Zhang Z, Mou Y, Hu Z (2008). Quantitative trait loci controlling Cu Ca Zn Mn and Fe content in rice grains. J Genet.

[CR87] Lu L, Tian S, Liao H, Zhang J, Yang X, Labavitch JM, Chen W (2013). Analysis of metal element distributions in rice (*Oryza sativa* L.) Seeds and relocation during germination based on x-ray fluorescence imaging of Zn, Fe, K, Ca, and Mn. PLoS One.

[CR88] Mabesa RL, Impa SM, Grewal D, Johnson-Beebout SE (2013). Contrasting grain-Zn response of biofortification rice (*Oryza sativa* L) breeding lines to foliar Zn application. Field Crop Res.

[CR89] Maret W, Sandstead HH (2006). Zinc requirements and the risks and benefits of zinc supplementation. J Trace Elem Med Biol.

[CR90] Masuda H, Ishimaru Y, Aung MS, Kobayashi T, Kakei Y, Takahashi M, Higuchi K, Nakanishi H, Nishizawa NK (2012) Iron biofortification in rice by the introduction of multiple genes involved in iron nutrition. Sci Rep 2:543 10.1038/srep00543 PMC3408131 22848789

[CR91] Masuda H, Aung MS, Nishizawa NK (2013). Iron biofortification of rice using different transgenic approaches. Rice.

[CR92] McCouch SR, Zhao K, Wright M, Tung CW, Ebana K, Thomson M, Reynolds A, Wang D, DeClerck G, Ali MA (2010). Development of genome-wide SNP assays for rice. Breed Sci.

[CR93] Moraghan T, Sims A, Smith L (1999). Zinc in wheat grain as affected by nitrogen fertilization and available soil zinc. J Plant Nutr.

[CR94] Nagarathna TK, Shankar AG, Udayakumar M (2010). Assessment of genetic variation in zinc acquisition and transport to seed in diversified germplasm lines of rice (*Oryza sativa* L). J Agric Technol.

[CR95] Nagesh P, Babu VR, Usharani G, Reddy TD (2012). Heterosis studies for grain iron and zinc content in rice (*Oryza sativa* L). Annals of Biological Res.

[CR96] Nemeño GA, Sanchez PB, Badayos RB, Sta Cruz PC, Mamaril CP (2010). Effect of water management on zinc concentration in rice grains.

[CR97] Norton GJ, Deacon CM, Xiong L, Huang S, Meharg AA, Price AH (2010). Genetic mapping of the rice ionome in leaves and grain: identification of QTLs for 17 elements including arsenic cadmium iron and selenium. Plant Soil.

[CR98] Norton GJ, Douglas A, Lahner B, Yakubova E, Guerinot ML (2014). Genome wide association mapping of grain arsenic copper molybdenum and zinc in rice (*Oryza sativa* L) grown at four international field sites. PLoS One.

[CR99] Nozoye T, Nagasaka S, Kobayashi T, Takahashi M, Sato Y, Sato Y, Uozumi N, Nakanishi H, Nishizawa NK (2011). Phytosiderophore efflux transporters are crucial for iron acquisition in graminaceous plants. J Biol Chem.

[CR100] Ogo Y, Itai RN, Nakanishi H, Inoue H, Kobayashi T, Suzuki M, Takahashi M, Mori S, Nishizawa NK (2006). Isolation and characterization of *IRO2*, a novel iron-regulated bHLH transcription factor in graminaceous plants. J Exp Bot.

[CR101] Ogo Y, Itai RN, Nakanishi H, Kobayashi T, Takahashi M, Mori S, Nishizawa NK (2007). The rice bHLH protein *OsIRO2* is an essential regulator of the genes involved in Fe uptake under Fe-deficient conditions. Plant J.

[CR102] Ogo Y, Kobayashi T, Itai RN, Nakanishi H, Kakei Y, Takahashi M, Toki S, Mori S, Nishizawa NK (2008). A novel NAC transcription factor, IDEF2, that recognizes the iron deficiency-responsive element 2 regulates the genes involved in iron homeostasis in plants. J Biol Chem.

[CR103] Ogo Y, Itai RN, Kobayashi T, Aung MS, Nakanishi H, Nishizawa NK (2011). *OsIRO2* is responsible for iron utilization in rice and improves growth and yield in calcareous soil. Plant Mol Biol.

[CR104] Olsen LI, Palmgren MG (2014) Many rivers to cross: the journey of zinc from soil to seed. Front Plant Sci 5:30 10.3389/fpls.2014.00030 PMC3921580 24575104

[CR105] Ozturk L, Yazici MA, Yucel C, Torun A, Cekic C, Bagci A, Ozkan H, Braun HJ, Sayers Z, Cakmak I (2006). Concentration and localization of zinc during seed development and germination in wheat. Physiol Plant.

[CR106] Paltridge N, Palmer L, Milham P, Stangoulis J (2012). Energy-dispersive x-ray fluorescence analysis of zinc and iron concentration in rice and pearl millet grain. Plant and Soil.

[CR107] Paul S, Ali N, Gayen D, Datta SK, Datta K (2012). Molecular breeding of *Osfer2* gene to increase iron nutrition in rice grain. GM Crops Food.

[CR108] Petry N, Egli I, Gahutu JB, Tugirimana PL, Boy E, Hurrell R (2012). Stable iron isotope studies in Rwandese women indicate that the common bean has limited potential as a vehicle for iron biofortification. J Nutr.

[CR109] Pfeiffer WH, McClafferty B, Kang MS, Priyadarshan PM (2008). Biofortification: breeding micronutrient-dense crops. Breeding major food staples.

[CR110] Phattarakul N, Rerkasem B, Li LJ, Wu LH, Zou CQ, Ram H, Sohu VS, Kang BS, Surek H, Kalayci M, Yazici A, Zhang FS, Cakmak I (2012). Biofortification of rice grain with zinc through zinc fertilization in different countries. Plant Soil.

[CR111] Prasad AS (2004). Zinc deficiency: its characterization and treatment. Met Ions Biol Syst.

[CR112] Priya SN, Sarla N, Ramannan R (2015). Expression analysis of candidate genes present in the QTL regions for both iron and zinc in the F_7_ RILs of Madhukar x Swarna. International conference on Transcriptomics. Transcriptomics.

[CR113] Prom-u-thai C, Dell B, Thomson G, Rerkasem B (2003). Easy and rapid detection of iron in rice grain. Sci Asia.

[CR114] Rai KN, Govindaraj M, Rao AS (2012). Genetic enhancement of grain iron and zinc content in pearl millet. Qual. Assur. Saf. Crops Food.

[CR115] Ramesh SA, Shin R, Eide DJ, Schachtman P (2003). Differential metal selectivity and gene expression of two zinc transporters from rice. Plant Physiol.

[CR116] Ren XL, Liu QL, Wu DX, Shu QY (2006). Variations in concentration and distribution of health-related elements affected by environmental and genotypic differences in rice grains. Rice Science.

[CR117] Rengel Z, Batten GD, Crowley DE (1999) Agronomic approaches for improving the micronutrient density in edible portions of field crops. Field Crop Res 60:27–40

[CR118] Ricachenevsky FK, Sperotto RA, Menguer PK, Sperb ER, Lopes KL, Janette P, Fett JP (2011). Zinc-induced facilitator-like family in plants: lineage-specific expansion in monocotyledons and conserved genomic and expression features among rice (*Oryza sativa*) paralogs. BMC Plant Biol.

[CR119] Roohani N, Hurrell R, Kelishadi R, Schulin R (2013). Zinc and its importance for human health: An integrative review. J Res Med Sci.

[CR120] Rose TJ, Impa SM, Rose MT, Pariasca-Tanaka J, Mori A, Heuer S, Johnson-Beebout SE, Wissuwa M (2013). Enhancing phosphorus and zinc acquisition efficiency in rice: a critical review of root traits and their potential utility in rice breeding. Ann Bot.

[CR121] Sadeghzadeh B (2013). A review of zinc nutrition and plant breeding. J Soil Sci Plant Nutr.

[CR122] Sala M, CR A k, Geetha S, Gnanamalar RP, Hemalatha G (2013). Variability studies for iron and zinc content on segregating population of rice. Electronic J Plant Breed..

[CR123] Samak A, Hittalamani H, Shashidhar HE, Biradar H (2011). Studies on genetic variability and genetic control for protein and micronutrient content in F_4_ and F_5_ generation of rice (*Oryza Sativa* L). Asian J. Plant Sci..

[CR124] Sarla N, Swamy BPM, Kaladhar K, Anuradha K, Rao VY, Batchu AK, Agarwal S, Babu AP, Sudhakar T, Sreenu K, Longvah T, Surekha K, Rao KV, Ashoka Reddy G, Roja TV, Kiranmayi SL, Radhika K, Manorama K, Cheralu C, Viraktamath BC (2012). Increasing iron and zinc in rice grains using deep water rices and wild species – identifying genomic segments and candidate genes. Qual. Assur. Saf. Crops Food.

[CR125] Sasaki A, Yamaji N, Xia J, Ma JF (2011). *OsYSL6* is involved in the detoxification of excess manganese in rice. Plant Physiol.

[CR126] Sasaki A, Yamaji N, Yokosho K, Ma JF (2012). Nramp5 is a major transporter responsible for manganese and cadmium uptake in rice. Plant Cell.

[CR127] Sasaki A, Yamaji N, Ma JF (2014). Over expression of *OsHMA3* enhances Cd tolerance and expression of Zn transporter genes in rice. J Exp Bot.

[CR128] Sathisha TN (2013) Genetic variation among traditional landraces of rice with specific reference to nutrition al quality. Karnataka J Agric Sci 26:474

[CR129] Shahzad Z, Rouached H, Rakha A (2014). Combating mineral malnutrition through iron and zinc biofortification of cereals. Compr. Rev. Food Sci. Food Saf..

[CR130] Sharifi P (2013). Genetic analysis of nutritional quality traits in milled kernels of hybrid rice based on the additive-dominance model. J Trop Agric and Fd Sc.

[CR131] Sharma A, Patni B, Shankhdhar D, Shankhdhar SC (2013). Zinc-an indispensable micronutrient. Physiol Mol Biol Plants.

[CR132] Shi RL, Zhang YQ, Chen XP, Sun QP, Zhang FS, Römheld V, Zou CQ (2010). Influence of long-term nitrogen fertilization on micronutrient density in grain of winter wheat (*Triticum aestivum* L). J Cereal Sci.

[CR133] Shivay YS, Kumar D, Prasad R (2008). Effect of zinc-enriched urea on productivity zinc uptake and efficiency of an aromatic rice-wheat cropping system. Nutr Cycl Agro ecosyst.

[CR134] Singh MK, Prasad SK (2014). Agronomic aspects of zinc biofortification in rice (*Oryza sativa* L). Proc Natl Acad Sci India Sect B Biol Sci.

[CR135] Singh N, Jayaswal PK, Panda K, Mandal P, Kumar V, Singh B, Mishra S, Singh Y, Renu Singh R, Rai V, Gupta A, Sharma TR, Singh NK (2015). Single-copy gene based 50 K SNP chip for genetic studies and molecular breeding in rice. Sci Rep.

[CR136] Slamet-Loedin IH, Johnson-Beebout SE, Impa S, Nikolaos T (2015). Enriching rice with Zn and Fe while minimizing Cd risk. Front Plant Sci.

[CR137] Sperotto RA, Ricachenevsky FK, Waldow V, Müller ALH, Dressler VL, Fett JP (2013). Rice grain Fe, Mn and Zn accumulation: How important are flag leaves and seed number?. Plant Soil Environ..

[CR138] Sperotto RA, Boff T, Duartea GL, Santos LS, Grusakc MA, Fett JP (2010). Identification of putative target genes to manipulate Fe and Zn concentrations in rice grains. J Plant Physiol.

[CR139] Stangoulis J, Sison C (2008) Crop Sampling Protocols for Micronutrient Analysis. HarvestPlus Technical Monograph Series 7, ISBN 978-0-9818176-0-6

[CR140] Stangoulis J, Huynh BL, Welch RM, Choi EY, Graham RD (2007). Quantitative trait loci for phytate in rice grain and their relationship with grain micronutrient content. Euphytica.

[CR141] Stein RJ, Ricachenevsky FK, Fett JP (2009). Differential regulation of the two rice ferritin genes (*OsFER1* and *OsFER2*). Plant Sci.

[CR142] Stomph T, Jiang W, Struik PC (2009). Zinc biofortification of cereals: rice differs from wheat and barley. Trends in Plant Sci.

[CR143] Stomph TJ, Jiang W, Van Der Putten PE, Struik PC (2014). Zinc allocation and re-allocation in rice. Front Plant Sci.

[CR144] Subedi P, Shrestha J (2015). Improving soil fertility through Azolla application in low land rice:A review. Azarian J Agriculture.

[CR145] Susanto U (2008). Mapping of quantitative trait loci for high iron and zinc content in polished rice (*Oryza sativa* L) grain and some agronomic traits using simple sequence repeats markers.

[CR146] Swamy BPM, Kumar A (2013b) Genomics-based precision breeding approaches to improve drought tolerance in rice. Biotechnol Adv 31:1308–1318 10.1016/j.biotechadv.2013.05.004 23702083

[CR147] Swamy BPM, Kaladhar K, Anuradha K, Batchu AK, Longvah T, Viraktamath BC, Sarla N (2011). Enhancing iron and zinc concentration in rice grains using wild species.

[CR148] Swamy BPM, Kaladhar K, Sarla N, Shoba Rani N, Prasad GSV, Viraktamath BC (2012). Genome wide mapping of quantitative trait loci for grain quality traits in *O. sativa* cv Swarna x *O. nivara* backcross population. J Heredity.

[CR149] Swamy BPM, Ahmed HU, Henry A, Dixit S, Vikram P, Ram T, Verulkar SV, Perraju P, Mandal NP, Variar M, Mishra KK, Lata TA, Karmakar B, Mauleon R, Satoh K, Moumeni A, Kikuchi S, Leung H, Kumar A (2013a) Genetic, physiological, and gene expression analyses reveal multiple QTLs that enhance yield of rice mega-variety IR64 under drought. PLoS One 8:e62795 10.1371/journal.pone.0062795 PMC3648568 23667521

[CR150] Swamy BPM, Kaladhar K, Ashok Reddy G, Viraktamath BC, Sarla N (2014). Mapping and introgression QTLs for yield and related traits in two backcross populations derived from *O. sativa* cv Swarna and two accessions of *O. nivara*. J Genet.

[CR151] Swamy BPM, Inabangan-Asilo MA, Amparado A, Manito C, Reinke R (2015). Progress in development of high grain Zinc rice varieties for Asia.

[CR152] Takahashi M, Nozoye T, Kitajima N, Fukuda N, Hokura A, Terada Y, Nakai I, Ishimaru Y, Kobayashi T, Nakanishi H, Naoko K, Nishizawa NK (2009). In vivo analysis of metal distribution and expression of metal transporters in rice seed during germination process by microarray and X-ray Fluorescence Imaging of Fe, Zn, Mn, and Cu. Plant Soil.

[CR153] Takahashi R, Ishimaru Y, Ogo HU, Senoura T, Nishizawa NK, Nakanishi H (2012). The *OsHMA2* transporter is involved in root-to-shoot translocation of Zn and Cd in rice. Plant Cell Environ..

[CR154] Tan S, Han R, Li P, Yang G, Li S, Zhang P, Wang WB, Zhao WZ, Yin LP (2015). Over-expression of the *MxIRT1* gene increases iron and zinc content in rice seeds. Transgenic Res.

[CR155] Tariq M, Hameed S, Malik KA, Fauzia YH (2007). Plant root associated bacteria for zinc mobilization in rice. Pak J Bot.

[CR156] Thongbam PD, Tarentoshi, Raychaudhury M, Durai A, Das SP, Ramesh T, Patiram, Ramya KT, Fiyaz RA, Ngachan SV (2012). Studies on grain and food quality traits of some indigenous rice cultivars of North-eastern Hill Region of India. J Agric Sci.

[CR157] Thorne-Lyman AL, Valpiani N, Sun K, Semba RD, Klotz CL, Kraemer K, Akhter N, de Pee S, Moench-Pfanner R, Sari M, Bloem MW (2010). Household dietary diversity and food expenditures are closely linked in rural Bangladesh increasing the risk of malnutrition due to the financial crisis. J. Nutr..

[CR158] Trijatmiko KR, Dueñas C, Tsakirpaloglou N, Torrizo L, Arines FM, Adeva C, Balindong J, Oliva N, Sapasap MV, Borrero J, Rey J, Francisco P, Nelson A, Nakanishi H, Lombi E, Tako E, Glahn RP, Stangoulis J, Chadha-Mohanty P, Johnson AAT, Joe Tohme J, Barry G, Slamet-Loedin IH (2016). Biofortified indica rice attains iron and zinc nutrition dietary targets in the field. Sci Rep.

[CR159] Vaid SK, Kumar B, Sharma A, Shukla AK, Srivastava PC (2014). Effect of Zn solubilizing bacteria on growth promotion and Zn nutrition of rice. J Soil Sci Plant Nutr.

[CR160] Varshney RK, Nayak SN, May GD, Jackson SA (2009). Next generation sequencing technologies and their implications for crop genetics and breeding. Trends Biotechnol.

[CR161] Vasconcelos MW, Li GW, Lubkowitz MA, Grusak MA (2008). Characterization of the PT clade of oligopeptide transporters in rice. Plant Genome.

[CR162] Velu G, Bhattacharjee R, Rai KN, Sahrawat KL, Longvah T (2008) A simple and rapid screening method for grain zinc content in pearl millet. J SAT Agric Res

[CR163] Velu G, Ortiz-Monasterio I, Cakmak I, Hao Y, Singh RP (2013). Biofortification strategies to increase grain zinc and iron concentrations in wheat. J. Cereal Sci..

[CR164] Wang LC, Busbey S (2005). Images in clinical medicine acquired *Acrodermatitis enteropathica*. N Engl J Med.

[CR165] Wang Y, Wei Y, Dong L, Lu L, Feng Y, Zhang J, Pan F, Yang F (2014). Improved yield and Zn accumulation for rice grain by Zn fertilization and optimized water management. Zhejiang Univ-Sci B.

[CR166] Waters BM, Sankaran RP (2011). Moving micronutrients from the soil to the seeds: genes and physiological processes from a biofortification perspective. Plant Sci.

[CR167] Waters BM, Uauy C, Dubcovsky J, Grusak MA (2009). Wheat (*Triticum aestivum*) NAM proteins regulate the translocation of iron, zinc, and nitrogen compounds from vegetative tissues to grain. J Exp Bot.

[CR168] Wei Y, Shohag MJ, Yang X (2012). Biofortification and bioavailability of rice grain zinc as affected by different forms of foliar zinc fertilization. PLoS One.

[CR169] Welch RM, Graham RD (2004). Breeding for micronutrients in staple food crops from a human nutrition perspective. J Exp Bot.

[CR170] Wessells KR, Brown KH (2012). Estimating the global prevalence of Zinc deficiency: results based on zinc availability in national food supplies and the prevalence of stunting. PLoS One.

[CR171] White PJ, Broadley MR (2011). Physiological limits to zinc biofortification of edible crops. Front Plant Sci.

[CR172] Widodo B, Broadley MR, Rose T, Frei M, Pariasca-Tanaka J, Yoshihashi T, Thomson M, Hammond JP, Aprile A, Close TJ, Ismail AM, Matthias Wissuwa M (2010). Response to zinc deficiency of two rice lines with contrasting tolerance is determined by root growth maintenance and organic acid exudation rates, and not by zinc-transporter activity. New Phytologist.

[CR173] Wissuwa M, Ismail AM, Yanagihara S (2006). Effects of zinc deficiency on rice growth and genetic factors contributing to tolerance. Plant Physiol.

[CR174] Wissuwa M, Ismail AM, Graham RD (2007). Rice grain zinc concentrations as affected by genotype native soil-zinc and zinc fertilization. Plant Soil.

[CR175] Wissuwa M, Ismail AM, Robin D, Graham RD (2008). Rice grain zinc concentrations as affected by genotype native soil-zinc availability and zinc fertilization. Plant Soil.

[CR176] Wu CY, Lu LL, Yang XE, Geng Y, Wei YY, Hao HL, Stoffella PJ, He ZL (2010). Uptake translocation and remobilization Zinc absorbed at different growth stages by rice genotypes of different Zn densities. J Agric Food Chem.

[CR177] Yamaguchi N, Ishikawa S, Abe T, Baba K, Arao T, Terada Y (2012). Role of the node in controlling traffic of cadmium zinc and manganese in rice. J Exp Bot.

[CR178] Yamaji N, Xia J, Mitani-Ueno N, Yokosho K, Feng Ma J (2013). Preferential delivery of zinc to developing tissues in rice is mediated by P-type heavy metal ATPase *OsHMA2*. Plant Physiol.

[CR179] Yang W, Liu Y, Chen L, Gao T, Hu B, Zhang D, Liu F (2007). Zinc Uptake regulator (*zur*) Gene involved in zinc homeostasis and virulence of *Xanthomonas oryzae pv. Oryzae*. Rice Current Microbiology.

[CR180] Yang X, Huang J, Jiang Y, Zhang HS (2009). Cloning and functional identification of two members of the ZIP (Zrt Irt-like protein) gene family in rice (*Oryza sativa* L). Mol Biol Rep.

[CR181] Yang Z, Wu Y, Li Y, Ling H, Chu C (2009). *OsMT1a*, a type 1 metallothionein, plays the pivotal role in zinc homeostasis and drought tolerance in rice. Plant Mol Biol.

[CR182] Yin HJ, Gao XP, Stomph T, Li L, Zhang F, Zou CQ (2016). Zinc concentration in rice (*Oryza sativa* L.) grains and allocation in plants as affected by different zinc fertilization strategies. Commun. Soil Sci. Plant Anal..

[CR183] Yokosho K, Yamaji N, Ueno D, Mitani N, Ma JF (2009). *OsFRDL1* is a citrate transporte required for efficient translocation of iron in rice. Plant Physiol.

[CR184] Yuan L, Yang S, Liu B, Zhang M, Wu K (2012). Molecular characterization of a rice metal tolerance protein, *OsMTP1*. Plant Cell Rep.

[CR185] Zarcinas BA, Cartwright B, Spouncer LR (1987). Nitric acid digestion and multi-element analysis of plant material by inductively coupled plasma spectrometry. Commun Soil Sci Plant Anal.

[CR186] Zhang MW, Peng ZM, Du YQ (1996). Combining ability and stability analysis of the content of trace elements Fe Zn and Mn in special rice grains. Chinese J Rice Sci.

[CR187] Zhang MW, Guo BJ, Peng ZM (2004). Genetic effects on Fe Zn Mn and P contents in *Indica* black pericarp rice and their genetic correlations with grain characteristics. Euphytica.

[CR188] Zhang J, Wu LH, Wang MY (2008). Iron and zinc biofortification in polished rice and accumulation in rice plant (*Oryza sativa* L) as affected by nitrogen fertilization. Acta Agricul Scand B.

[CR189] Zhang X, Zhang G, Guo L, Wang H, Zeng D, Dong G, Qian Q, Xue D (2011). Identification of quantitative trait loci for Cd and Zn concentrations of brown rice grown in Cd-polluted soils. Euphytica.

[CR190] Zhang Y, Xu Y, Yi H, Gong J (2012). Vacuolar membrane transporters *OsVIT1* and *OsVIT2* modulate iron translocation between flag leaves and seeds in rice. Plant J.

[CR191] Zhang M, Pinson SRM, Tarpley L, Huang X, Lahner B (2014). Mapping and validation of quantitative trait loci associated with concentration of 16 elements in un milled rice grain. Theor Appl Genet.

