# Supplementary material for: Advances in breeding for high grain Zinc in Rice
Source: Rice (N Y). 2016 Sep 26;9:49. doi: 10.1186/s12284-016-0122-5 (PMC5037106; doi:10.1186/s12284-016-0122-5)
Supplement: Additional file 1: Table S1. — List of Zinc homeostasis genes and their putative functions Chen et al. 2008; Dräger et al. 2004; Gross et al. 2003; Grotz & Guerinot 2006; Kobayashi et al. 2003; Kobayashi et al. 2005; Koike et al. 2004; Lee et al. 2010a; Lee et al. 2010b; Ricachenevsky et al. 2011; Sasaki et al. 2012; Stein et al. 2009; Takahashi et al. 2012; Vasconcelos et al. 2008; Yang et al. 2007; Yang et al. 2009a; Yang et al. 2009b; Yokosho et al. 2009; Yuan et al. 2012. (DOC 44 kb) [file 12284_2016_122_MOESM1_ESM.doc]

Additional file 1: **Table S1**. List of Zinc homeostasis genes and their putative functions

| Zinc homeostasis genes | Function | Reference |
| --- | --- | --- |
| *OsIRT1, OsIRT2* | Zn transporters | Lee and An 2009; Yang et al. 2009; Waters and Sankaran 2011 |
| *OsZIP1, OsZIP3, OsZIP4, OsZIP5, OsZIP7a, OsZIP8, OsZIP9, OsZIP10, OsZIP11* | Zn transporters | Ramesh et al. 2003; Gortz and Guerinot 2006; Chen et al. 2008; Lee et al. 2010a,2010b; Ishimaru et al. 2011; Waters and Sankaran 2011 |
| *OsOZT1* | Vacuolar Zn transporter | Lan et al. 2013 |
| *OsNAS1 ,OsNAS2 , OsNAS3,* | Phytosiderophores secretion, chelator, Zn uptake, tansport, loading | Inoue et al. 2003; Johnson et al. 2011, Lee et al.2011 |
| *OsTOM1* | Tansport of Phytosiderophores | Nozoy et al. 2011 |
| *OsHMA2,OsHMA3* | Zn uptake , translocation and loading | Takahashi et al.2012; Yamaji et al. 2013; Sasaki et al. 2014 |
| *OsMT1a* | Zn homeostasis | Yang et al. 2009 |
| *zur* | Zn homeostasis | Yang et al. 2007 |
| *OsNRAMP1, OsNRAMP4, OsNRAMP5, OsNRAMP6, OsNRAMP7, OsNRAMP8* | Transmembrane transport of Zn | Banerjee et al. 2011; Sasaki et al.2012; Agarwal et al. 2014 |
| *OsYSL2, OsYSL4, OsYSL6, OsYSL7, OsYSL8, OsYSL9, OsYSL12, OsYSL14, OsYSL15, OsYSL18* | pholem transport of metals, long distance transport of metals | Koike et al.2004; Lee et al..2009; Aoyama et al. 2009; Ishimaru et al. 2010; Sasaki et al. 2011; Inoue et al. 2009. Kakei et al. 2012 |
| *OsNAC* | Transcription factor inducing metal transporter genes | Banerjee et al. 2011; Gande et al.2014 |
| *NAM-B1* | Transcription factor inducing metal transporter genes | Waters et al. 2009 |
| *OsVIT1, OsVIT2* | Zn transport across tonoplast | Banerjee and Chandel 2011; Zhang et al. 2012 |
| *OsNAAT1, OsNAAT4,* | Nicotianamine biosynthesis | Inoue et al. 2008 |
| *OsDMAS1* | synthesis of mugineic acid | Bashir et al.2006 |
| *OsMTP1, OsMTP3* | Metal transporters | Drager et al. 2004; Yuan et al. 2012 |
| *OsFER1, OsFER2* | Chelator for Fe and Zn accumulation in grains | Stein et al. 2009; Paul et al. 2012 |
| *OsFRO2* | Transmembrane transport of Fe and Zn | Gross et al. 2003; Banerjee et al. 2011 |
| *OsZIFL2, OsZIFL3, OsZIFL4, OsZIFL5, OsZIFL7, OsZIFL9, OsZIFL10, OsZIFL12* | Zn homeostasis | Ricachenevsky et al. 2011 |
| *OsSAMS1, OsSAMS2* | Mugineic Acid biosynthesis | Widodo et al. 2010 |
| *OsIDEF1, OsIDEF2* | Transcription factor inducing metal transporter genes | Kobayashi et al. 2003,2005; Ogo et al. 2008 |
| *OsIRO2* | Transcription factor regulating Fe uptake | Ogo et al. 2006, 2007 |
| *OsFRDL1* | Fe transporter in xylem | Yokosho et al. 2009 |
| *OsOPT1, OsOPT3, OsOPT4, OsOPT5, OsOPT7* | Metal transporters found in almost every tissue | Vasconcelos et al. 2008 |
